# A preclinical randomized controlled multi-centre trial of anti-interleukin-17A treatment for acute ischaemic stroke

**DOI:** 10.1093/braincomms/fcad090

**Published:** 2023-03-23

**Authors:** Mathias Gelderblom, Simon Koch, Jan-Kolja Strecker, Carina Jørgensen, Lidia Garcia-Bonilla, Peter Ludewig, Ines Sophie Schädlich, Marius Piepke, Karoline Degenhardt, Christian Bernreuther, Hans Pinnschmidt, Thiruma V Arumugam, Götz Thomalla, Cornelius Faber, Jan Sedlacik, Christian Gerloff, Jens Minnerup, Bettina H Clausen, Josef Anrather, Tim Magnus

**Affiliations:** Department of Neurology, University Medical Center Hamburg-Eppendorf, 20246 Hamburg, Germany; Department of Neurology, University Medical Center Hamburg-Eppendorf, 20246 Hamburg, Germany; Department of Neurology with Institute of Translational Neurology, University of Münster, 48149 Münster, Germany; Department of Neurobiology Research, Institute of Molecular Medicine, University of Southern Denmark, 5000 Odense, Denmark; Feil Family Brain and Mind Research Institute, Weill Cornell Medicine, New York, NY 10065, USA; Department of Neurology, University Medical Center Hamburg-Eppendorf, 20246 Hamburg, Germany; Department of Neurology, University Medical Center Hamburg-Eppendorf, 20246 Hamburg, Germany; Department of Neurology, University Medical Center Hamburg-Eppendorf, 20246 Hamburg, Germany; Department of Neurology, University Medical Center Hamburg-Eppendorf, 20246 Hamburg, Germany; Department of Neuropathology, University Medical Center Hamburg-Eppendorf, 20246 Hamburg, Germany; Institute of Medical Biometry and Epidemiology, University Medical Center Hamburg-Eppendorf, 20246 Hamburg, Germany; Department of Physiology, Anatomy & Microbiology School of Life Sciences, La Trobe University, Melbourne 3086, Australia; Department of Neurology, University Medical Center Hamburg-Eppendorf, 20246 Hamburg, Germany; Translational Research Imaging Center, Clinic of Radiology, University of Münster, 48149 Münster, Germany; Department of Biomedical Engineering, Centre for the Developing Brain, School of Biomedical Engineering & Imaging Sciences, King's College London, London WC2R 2LS, UK; Department of Neurology, University Medical Center Hamburg-Eppendorf, 20246 Hamburg, Germany; Department of Neurology with Institute of Translational Neurology, University of Münster, 48149 Münster, Germany; Department of Neurobiology Research, Institute of Molecular Medicine, University of Southern Denmark, 5000 Odense, Denmark; Feil Family Brain and Mind Research Institute, Weill Cornell Medicine, New York, NY 10065, USA; Department of Neurology, University Medical Center Hamburg-Eppendorf, 20246 Hamburg, Germany

**Keywords:** stroke, preclinical randomized controlled trial, interleukin-17A, inflammation

## Abstract

Multiple consensus statements have called for preclinical randomized controlled trials to improve translation in stroke research. We investigated the efficacy of an interleukin-17A neutralizing antibody in a multi-centre preclinical randomized controlled trial using a murine ischaemia reperfusion stroke model. Twelve-week-old male C57BL/6 mice were subjected to 45 min of transient middle cerebral artery occlusion in four centres. Mice were randomly assigned (1:1) to receive either an anti-interleukin-17A (500 µg) or isotype antibody (500 µg) intravenously 1 h after reperfusion. The primary endpoint was infarct volume measured by magnetic resonance imaging three days after transient middle cerebral artery occlusion. Secondary analysis included mortality, neurological score, neutrophil infiltration and the impact of the gut microbiome on treatment effects. Out of 136 mice, 109 mice were included in the analysis of the primary endpoint. Mixed model analysis revealed that interleukin-17A neutralization significantly reduced infarct sizes (anti-interleukin-17A: 61.77 ± 31.04 mm^3^; IgG control: 75.66 ± 34.79 mm^3^; *P* = 0.01). Secondary outcome measures showed a decrease in mortality (hazard ratio = 3.43, 95% confidence interval = 1.157–10.18; *P* = 0.04) and neutrophil invasion into ischaemic cortices (anti-interleukin-17A: 7222 ± 6108 cells; IgG control: 28 153 ± 23 206 cells; *P* < 0.01). There was no difference in Bederson score. The analysis of the gut microbiome showed significant heterogeneity between centres (*R* = 0.78, *P* < 0.001, *n* = 40). Taken together, neutralization of interleukin-17A in a therapeutic time window resulted in a significant reduction of infarct sizes and mortality compared with isotype control. It suggests interleukin-17A neutralization as a potential therapeutic target in stroke.

## Teaser

To bridge the gap between experimental research and human clinical trials, Gelderblom *et al*. investigated the efficacy of interleukin-17A neutralization in a preclinical randomized controlled stroke trial. In the murine stroke model, neutralization of interleukin-17A resulted in a significant reduction of infarct sizes three days after ischaemia.

## Graphical Abstract

**Graphical Abstract:**
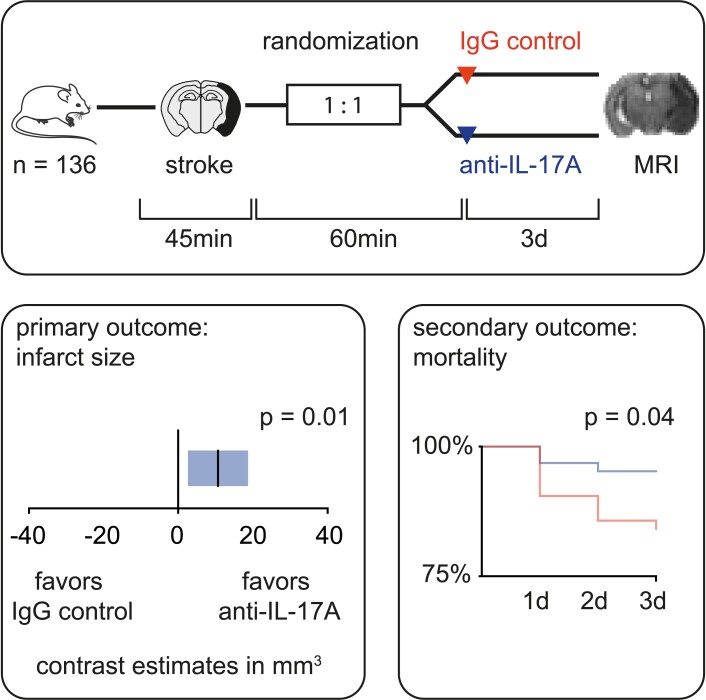

## Introduction

In the Western world, stroke is the leading cause of long-term disability and the third leading cause of death.^1^ Currently, treatment opportunities are limited to thrombolysis and thrombectomy which are only available to a subset of acute stroke patients. Furthermore, a significant proportion of stroke patients still suffers from clinically relevant neurological long-term deficits despite successful recanalization. Novel therapeutic strategies which are targeting ischaemic brain injury are urgently needed but still not available as the translation of experimental neuroprotective therapies from preclinical research into patient care has not been successful to date.^2^ A main reason for the lack in translation is the inappropriate design of preclinical stroke studies.^2-4^ Accordingly, neuroprotective compounds that have shown beneficial effects in murine experimental stroke failed in human clinical trials.^5,6^ These translational difficulties have resulted in the recommendations of The Stroke Therapy Academic Industry Roundtable and a recently updated version of the Animal Research: Reporting of In Vivo Experiments (ARRIVE) guidelines (ARRIVE 2.0).^7^ A broad range of scientists have supported and extended these guidelines with calls for rigorous study designs including preclinical randomized controlled trials (pRCT) to improve drug development and selection of potential candidates for clinical studies.^8,9^ Operational and statistical guidelines for multi-centre pRCT include: (i) harmonized experimental protocols; (ii) sample size calculation; (iii) randomization; (iv) blinding; (v) cross-validation and (vi) centralized study organization. With these demands, the organization and financing of pRCTs is complex. Furthermore, publication is challenging, since pRCTs are mostly confirmatory studies investigating already known compounds instead of novel therapeutic targets. Therefore, only two such trials have been published in experimental stroke research.^10,11^

Recent immunological research has indicated that inflammatory mechanisms are central to the pathophysiology of stroke.^12^ In particular, the highly conserved pro-inflammatory cytokine Interleukin-17A (IL-17A) holds key functions in the initiation of the excessive detrimental inflammatory response in the ischaemic brain in the first hours and days.^13,14^ In stroke, γδ T cells are the main source of IL-17A. These innate-like lymphocytes rapidly infiltrate and produce IL-17A in response to IL-1 and IL-23, with a peak of IL-17A expression between 48 and 72 h post-stroke.^15,16^ The main effect of IL-17A is the local induction of the early detrimental neutrophil infiltration into the ischaemic brain. Beside stroke, IL-17A is also implicated in the pathology of several autoimmune diseases such as rheumatoid arthritis, and psoriasis which led to Food and Drug Administration approved anti-IL-17A treatments.^17^ In murine models of experimental stroke, evidence for a detrimental role of IL-17A was seen in multiple single-centre studies.^13,14,18-20^ While these data support an important role of IL-17A in stroke, single-centre studies are biased by site-specific confounders, including animal housing conditions, the microbiome, the experimental set-up and even the investigators.

Therefore, we have performed a pRCT on IL-17A neutralization in experimental stroke. This study was carried out in Odense (Denmark), New York (USA), Hamburg (Germany) and Münster (Germany) using the transient middle cerebral artery occlusion model (tMCAO). Following a predefined protocol including randomization and blinding, application of mouse-monoclonal anti-IL-17A antibodies significantly reduced infarct size and mortality and resulted in less cortical neutrophil infiltration. This pRCT showed that neuralization of IL-17A is effective in a large sample size and across different study centres.

## Materials and methods

### Study design

The study was performed between 2017 (initiation) and 2018 (unblinding) in an international consortium consisting of four test centres. Infarct size on Day 3 after stroke was defined as the primary endpoint. Secondary endpoints were mortality, neurological outcome and neutrophil infiltration into the ischaemic hemisphere. Primary and secondary efficacy outcomes were assessed in the per-protocol population, which included all mice that completed the trial until Day 3. The exclusion criteria and any experimental details were predefined in the study protocol **(**Supplementary Figs 1 and 2**)**. The Hamburg study centre (M.G.) designed the study protocol, coordinated the trial and performed central data analysis. Hamburg (M.G.), New York (J.A.), Odense (B.H.C.) and Münster (J.M.) performed the tMCAO experiments. Sample size was determined *a priori* by performing a power analysis (Power = 0.95, G*Power software) with a targeted effect size of 1.2, based on previously published data on anti-IL-17A treatment.^14^ We conducted all experiments according to the Guide for the Care and Use of Laboratory Animals published by the US National Institutes of Health (publication No. 83-123, revised 1996) and performed all procedures in accordance with the ARRIVE guidelines. All animal experiments were approved by the respective local animal care committees.

### Mice

C57BL/6 mice were provided by different suppliers (Münster and Hamburg: Charles River, Odense: Taconic, New York: The Jackson Lab) and housed in the respective animal facility at least for 2 weeks before the experiments started. We used male mice with an age of 11–13 weeks and a bodyweight of 24–28 g. In total, 136 mice were randomized and treated. The animals were housed according to the local guidelines of each study centre and assessed at least once a day.

### Randomization and treatment

Mice were randomly assigned to treatment with either 500 µg of anti-IL-17A (3.33 μg/μl, clone MM17F3, eBioscience, RRID: AB_763585) or isotype IgG antibody (3.33 μg/μl, eBioscience, RRID: AB_470160) by the coordinating centre using a randomizer tool. Antibodies were injected intravenously 1 h after reperfusion. All participating surgeons, animal caretakers and researchers were blinded for the treatment groups.

### Transient middle cerebral artery occlusion

Transient middle cerebral artery occlusion was achieved with the intraluminal filament method (Doccol, Cat# 602112PK10) for 45 min as previously described.^21^ Body temperature was kept at 36°C during surgery and all mice were monitored for cerebral blood flow with the use of transcranial temporal laser Doppler. Only mice with a reduction of at least 80% in cerebral blood flow in the left middle cerebral artery (MCA) territory compared with the contralateral side were included in the study. After stroke induction, every mouse was repeatedly scored on a scale from 0 to 5 (0, no deficit; 1, preferential turning; 2, circling; 3, longitudinal rolling; 4, no movement and 5, death) immediately after re-awakening and every day until killing. Mice were sacrificed three days after tMCAO and perfused with 25 ml phosphate-buffered saline (10 ml/min) (PBS, SigmaAldrich, Cat# D8537-1L) and 25 ml 4% paraformaldehyde (10 ml/min) (PFA, Morphisto, Cat# 11762.01000) afterwards. Brains were kept in 4% PFA at 4°C for 24 h, stored in PBS at 4°C, and sent to the core laboratory in Hamburg for further analysis. Magnetic resonance imaging (MRI) was performed 4–6 weeks after removal of the brains.

### Infarct volume assessment by magnetic resonance imaging

*Ex vivo* MRI of the brains from Odense, New York and Münster was performed on a 7 T system (ClinScan, Bruker) in Hamburg. The specimens from Hamburg were measured on a 9.4 T system (BioSpec, Bruker) in Münster. For MRI acquisition, brains were placed in 15 ml falcon tubes filled with PBS. The imaging protocol comprised an apparent diffusion coefficient (ADC) and diffusion-weighted images (DWI). Calculations of infarct volumes were performed on ADC images using ImageJ (NIH).^22^ Infarct sizes were corrected for brain oedema.^23^ Infarct volume analysis was performed by two independent and blinded raters from Hamburg whose results highly correlated **(**Supplementary Fig. 3**)**. All MRI scans and analysis files were stored on a central database.

### Immunohistochemistry

Mice from the study cohorts in Hamburg, New York and Odense were additionally used for the immunohistochemical analysis of neutrophil infiltration. Only mice with infarcts involving both cortical and striatal areas on MRI were randomly selected. Following MRI, the PFA fixed brains were embedded in paraffin and coronal sections of 10 µm thickness were collected every 400 μm. For the immunostaining of neutrophils, we performed antigen retrieval in 10 mM citrate buffer (pH 6.0) and blocked with normal rabbit serum (Vector Laboratories, Cat# S-5000, RRID: AB_2336619). Sections were incubated with Ly6G (clone 1A8, 1/500, Biolegend, Cat# 127602, RRID: AB_1089180) overnight at 4°C. Vectastain Elite ABC HRP kit (Vector Laboratories, Cat# PK-6104, RRID: AB_2336823) was used for visualization. For nuclear staining, Nuclear Fast Red (SigmaAldrich, Cat# N3020-100 ml) was added for 5 min at room temperature. Images were acquired on a digital slide scanner (Hamamatsu; Nano Zoomer). Manual cell counting was done with the NanoZoomer digital pathology viewer (Hamamatsu). Individual neutrophils on the entire slide were marked by hand to avoid double counts. Assignment to either the cortical or striatal area was assessed by colocalization to the respective anatomical structures. Total numbers of neutrophils were determined according to the following calculation: Total number of neutrophils = *N*(*n*_1_ + *n*_2_+…*n_i_*)×40 (*N* = sum of all counted neutrophils in the brain, *n_i_* = number of neutrophils counted on each slide*_i_* and 40 = number of slides of 10 µm thickness in the position 1…*i*). For each brain, neutrophils were counted on 15 slides. Images of representative neutrophils in both groups were taken with a transmission light microscope (Apotome, Zeiss) and a ×63 plan-apochromatic oil differential interference contrast (DIC) objective.

### Collection of faeces

We collected faeces of 10 mice in each centre for the analysis of the gut microbiome. All mice were housed for at least 2 weeks in the animal facility of the respective centre to adapt to local microbiota. Specimens were collected early in the morning directly from each mouse to avoid the loss of anaerobic bacteria. Immediately after collection, the faeces were homogenized in 96% ethanol to stop DNA degradation.

### Nucleic acid extraction

Following resuspension and homogenization of the faeces with the MSC-96000 Nasopharyngeal Sampling Kit (Miraclean Technology, # MSC-96000), nucleic acids (DNA and RNA) were extracted from 200 µl sample volume on an automated extraction system (QIAsymphony, Qiagen, Hilden) using the DSP Virus/Pathogen Midi Kit (Qiagen, # 937055), according to the manufacturer’s instructions.

### 16S rRNA amplicon library preparation, MiSeq sequencing and bioinformatic analysis

V3–V4 region 16S rRNA amplicon sequencing was performed as recently published.^24^ The following primers containing the Illumina adapter consensus sequence were used: F: 5′-TCGTCGGCAGCGTCAGATGTGTATAAGAGACAGCCTACGGGNGGCWGCAG-3 and R: 5-GTCTCGTGGGCTCGGAGATGTGTATAAGAGACAGGACTACHVGGGTATCTAATCC-3. Samples were multiplexed using the Illumina Nextera XT Index Kit and sequenced by 500 PE sequencing on the MiSeq platform. FastQC (Babraham Bioinformatics, Babraham Institute, UK) was used to determine the average quality scores of each sample before and after paired reads. The paired ends in each sample were joined, and all sequences <250 bp and/or with a Phred score <33 were discarded. Quality filtering was applied using Quantitative Insights Into Microbial Ecology (QIIME) 53 (at Phred ≥ Q20). We performed operational taxonomic unit (OTU) clustering and alpha- and beta-diversity analysis using QIIME 2.^25^ A chimaera filter was applied using USEARCH 8.1. All sequences were clustered based on 97% similarity to reference sequences. The reads that did not meet the 97% similarity criteria were clustered *de novo*. Taxonomy levels of representative sequences in the OTU table were assigned at 95% similarity based on the SILVA database. We calculated alpha diversity based on the total amount of OTUs. Analysis of beta-diversity statistics [analysis of similarities (ANOSIM)] was performed to determine if differences between the distributions of microbiota profiles were significant.

### Real-time quantitative PCR analysis of segmented filamentous bacteria

Real-time quantitative PCR (RT-qPCR) was performed on 7 ng of purified faecal DNA in three technical replicates. PCR was performed with Power SYBR Green PCR Master Mix (Thermofischer, Cat# 4368706) on a LightCycler® 96 Instrument (Roche) with the following primers: segmented filamentous bacteria (SFB) 736F: GACGCTGAGGCATGAGAGCAT, SFB 844R: GACGGCACGRATTGTTATTCA, and universal bacterial r16S gene primers 16S-V2-101F: AGYGGCGIACGGGTGAGTAA and 16S-V2-361R: CYIACTGCTGCCTCCCGTAG. Ct values of SFB amplicons were normalized to r16S gene Ct values by the ΔCt method and data are shown as fold change over SFB levels in mice from Odense as reference group (ΔΔCt method). Analysis was performed with Excel (Microsoft) and GraphPad Prism (GraphPad Software).

### Flow cytometry

The flow cytometric analysis of immune cell infiltration was performed in an additional cohort of mice subjected to 45 min of tMCAO in Hamburg. These mice were not part of the study cohort used for the analysis of the primary outcome parameter. Mice were sacrificed three days after tMCAO, followed by perfusion with PBS and removal of the brain. To ensure that only mice with infarcts involving cortical and striatal areas were included in the analysis, brains were cut into 1 mm thick sections. Next, we performed a vital staining using 2% (wt/vol) 2,3,5-triphenyl-2-hydroxy-tetrazolium chloride (TTC). Following TTC staining, the ipsilesional cortex was dissected from the striatum under a microscope and the two tissue samples were processed separately. The tissue was digested for 30 min at 37°C [1 mg/ml collagenase (SigmaAldrich, Cat# 11088793001) and 0.1 mg/ml DNAse I (Roche, Cat# 11284932001) in Dulbecco′s Modified Eagle′s Medium (DMEM) (Gibco, Cat# 41965-039)], and pressed through a 40 µm cell strainer. Cells were incubated with standard erythrocyte lysis buffer on ice and separated from myelin and debris by Percoll gradient (Cytiva Sweden, Cat# 17-5445-01) centrifugation and stained for 30 min at 4°C. The following antibodies were used in a dilution of 1:100: Biolegend: T-cell receptor γ/δ (PerCP-Cy5.5; clone GL3, Cat# 118118, RRID: AB_10612572), CD11b (PE-Cy7; M1/70, Cat# 101216, RRID: AB_312799), CD3 (BV421; 17A2, Cat# 100228, RRID: AB_2562553), B220 (BV570; RA3-6B2, Cat# 103237, RRID: AB_10900264), F4/80 (BV605; BM8, Cat# 123133, RRID: AB_2562305), CD8 (BV650; 53-6.7, Cat# 100742, RRID: AB_2563056), NK1.1 (BV710; PK136, Cat# 108745, RRID: AB_2563286), CD4 (BV785; RM4-5, Cat# 100551, RRID: AB_11218992), eBioscience: MHCII (FITC; M5/114.15.2, Cat# 11-5321-82, RRID: AB_465232), CD11c (APC; N418, Cat# 17-0114-82, RRID: AB_469346), CD45 (APC-eFluor 780; 30-F11, Cat# 47-0451-82, RRID: AB_1548781), BD Bioscience: Ly6G (PE; 1A8, Cat# 551461, RRID: AB_394208). For cell counting, BD Trucount tubes (BD Biosciences, Cat# 663028) were used. Fluorescence-activated cell sorting measurements were performed on an LSR Fortessa (BD Biosciences) in Hamburg. Data analysis was done with FlowJo (BD Biosciences).

### Statistical analysis

We computed a generalized linear-mixed model analysis (SPSS, IBM, © 2019) to test for treatment effects on the infarct volume. This model allowed us to account for two raters as random effects. Fixed effects were set as treatment and centre to compute pairwise contrasts between the treatment groups for each centre. Normal distribution was checked beforehand by histogram analysis. For the pairwise comparisons, *P*-values were adjusted with Šidák’s correction to account for multiple testing. Each specimen was divided into one of the two sub-groups (>30 mm^3^ versus < 30 mm^3^) by two independent raters. Within each sub-group, we conducted the same analysis as we did in the main group. Pearson’s *r* was calculated for inter-rater reliability. Mortality was analysed with a log-rank test. Bederson scores were analysed with a Mann–Whitney test for non-parametric data and bodyweight was analysed using a mixed model analysis to account for missing values with time points and treatments as fixed effects. Šidák’s test was assessed to test for pairwise contrasts of the two treatment groups on each time point. Flow cytometric data on cortical and striatal immune cell infiltration was analysed with a two-sided Student’s *t*-test for dependent samples. Neutrophil counts from immunohistochemical studies were analysed with a multivariate analysis of variances with repeated measurements of variances with compartments and treatments as independent variables. Šidák’s test was assessed to check for pairwise contrasts of the two treatments in the two compartments. Differences in the OTUs were assessed with *t*-tests for independent samples (QIIME 2). An analysis of similarities was performed to test for significance in the beta-diversity (QIIME 2). RT-qPCR data were analysed with Student’s *t*-tests for independent samples. In all tests, the alpha level was set at 0.05. If not stated otherwise GraphPad Prism 8 was used for the analysis.

### Data availability

The complete raw data (flow cytometry data, TTC staining, histology, MRI scans, microbiom data) is available under https://doi.org/10.25452/figshare.plus.c.6371439.

## Results

### Characteristics of the mice

**Figure 1:**
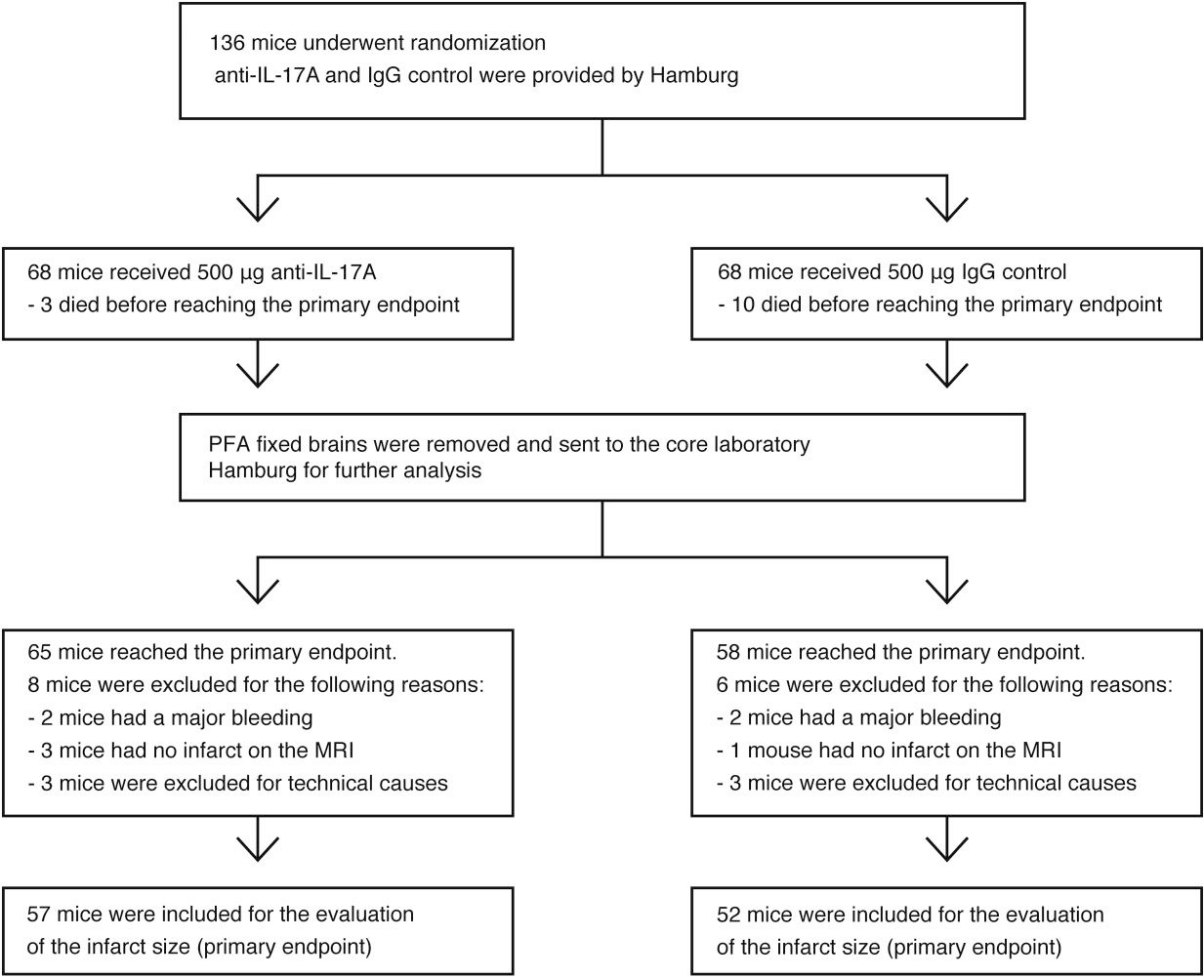
**Design and enrolment of the preclinical randomized multi-centre trial. Illustration of included and excluded animals.** Mice were treated either with 500 μg of anti-IL-17A or 500 μg of IgG control 1 h following reperfusion. Randomization, distribution of antibodies and blinded analysis of infarct volumes were performed by the core laboratory in Hamburg. Only brains of mice that survived until Day 3 were included in the final analysis of infarct volumes.

A total of 136 C57BL/6 mice were randomly assigned to treatment with either anti-IL-17A or isotype IgG antibody intravenously 1 h after tMCAO (45 min) in four independent research centres in Odense (Denmark), New York (USA), Hamburg, and Münster (Germany) **(**Fig. 1**)**. Coded antibodies and filaments were provided by Hamburg. All participating surgeons, animal caretakers and researchers were blinded for the treatment groups. Primary endpoint was infarct size on Day 3 in *ex vivo* MRI scans, secondary endpoints included mortality, neutrophil infiltration and the Bederson score. Unblinding was performed following completion of the statistical analyses. In the anti-IL-17A group, eight mice were excluded for the following reasons: major haemorrhage (*n* = 2), absence of infarction on the MRI (*n* = 3) or destruction of the brain during preparation (*n* = 3). In the isotype IgG control group, six mice were excluded for the following reasons: major haemorrhage (*n* = 2), undetectable infarct on the MRI (*n* = 1) or destruction of the brain during preparation (*n* = 3). The mortality until Day 3 was *n* = 3 in the IL-17A treatment group and *n* = 10 in the isotype control group. A total of 109 mice was included in the per-protocol analysis of the primary endpoint (IL-17A treatment group *n* = 57; isotype control group *n* = 52).

### Neutralization of interleukin-17A protects from ischaemic stroke and reduces mortality

**Figure 2:**
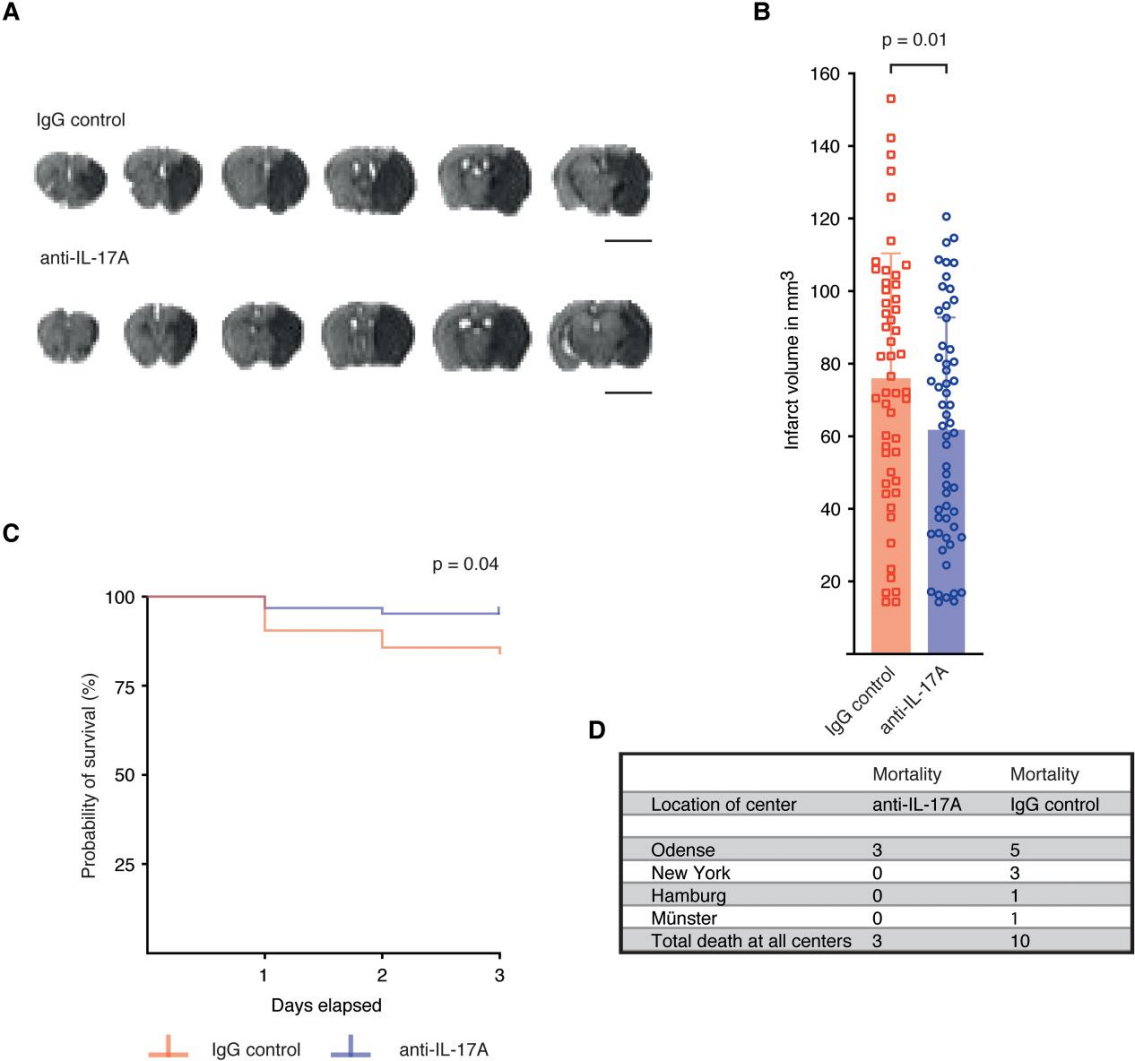
**Neutralization of IL-17A significantly reduced infarct size and mortality.** (**A)** Representative ADC weighted MRI images of mice treated with anti-IL-17A or IgG control (scale bar 5 mm). **(B)** Infarct volumes of both treatment groups from all centres (pooled data). Infarct volumes were analysed three days following tMCAO by MRI. The infarct volumes of rater one are shown in mm^3^. Graphs show mean ± SD. Statistical significance was assessed by linear-mixed model analysis (*F*(1,209) = 6.56, *P* = 0.01, *n* = 109). **(C, D)** Survival rate and number of dead mice. Statistical significance was assessed by a log-rank test (*χ*^2^_(1)_ = 4.15, *P* = 0.04, *n* = 123).

Infarct sizes were determined in a blinded approach by the volume of the ADC lesion on post-mortem MRI scans. Three days following tMCAO, the mean infarct volume and SD in the pooled data set was 61.77 ± 31.04 mm^3^ in the anti-IL-17A treatment group and 75.66 ± 34.79 mm^3^ in the IgG control group. To analyse the effects of IL-17A neutralization, we employed a linear-mixed model analysis on the study cohort with the two investigators as random effects, which revealed a significant reduction of infarct sizes in the anti-IL-17A treatment group compared with the IgG control group **(**Fig. 2A and B**)**. When analysing infarct sizes from single centres, we observed a significant effect in the Hamburg cohort (Supplementary Fig. 4A and B). Furthermore, mortality was significantly reduced in the anti-IL-17A group **(**Fig. 2C and D**)**. The probability to die was more than three times higher for mice in the IgG control group than in the anti-IL-17A group (hazard ratio = 3.43, 95% confidence interval = 1.157–10.18). Bederson scores **(**Supplementary Fig. 5A) and weight loss until Day 3 **(**Supplementary Fig. 5B**)** did not differ between groups.

### IL-17A neutralization reduces lesion size of large infarcts

**Figure 3:**
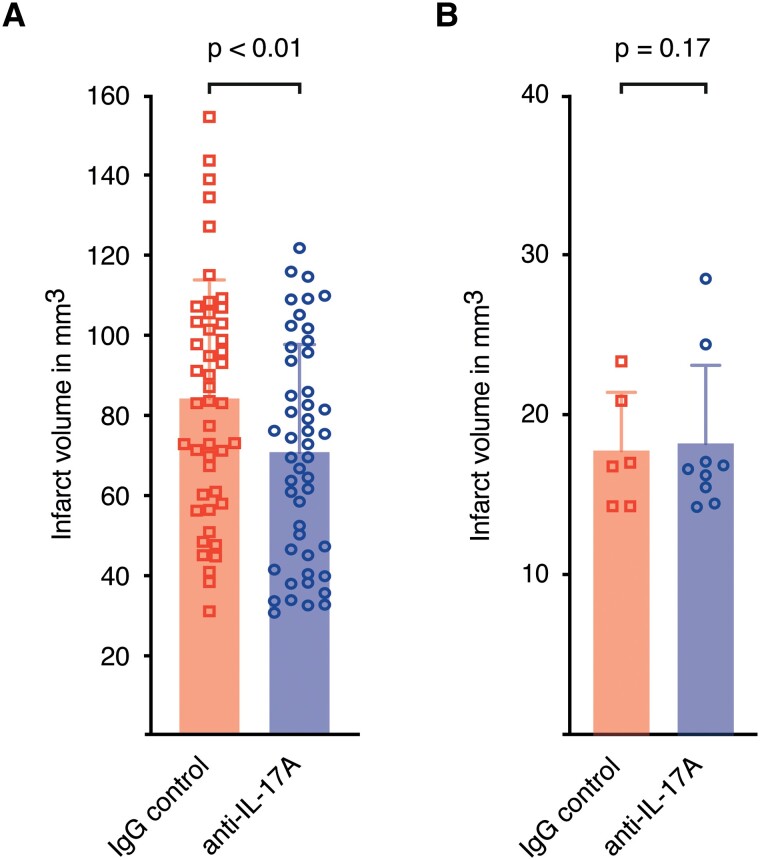
**IL-17A neutralization reduced the lesion size of large infarcts only.** (**A)** Analysis of infarct volumes > 30 mm^3^ of both treatment groups (pooled data set). Infarct volumes of rater one are shown in mm^3^. Graphs show mean ± SD. Statistical significance was assessed by linear-mixed model analysis (*F*(1,179) _=_ 8.17, *P* < 0.01, *n* = 94). **(B)** Analysis of infarct volumes < 30 mm^3^ of both treatment groups (pooled data set, *F*(1,22) _=_ 2.06, *P* = 0.17, *n* = 15).

Next, we analysed the effects of IL-17A neutralization on large infarcts (>30 mm^3^) with involvement of both cortical and striatal regions and small infarcts (<30 mm^3^), which are mainly affecting the striatum only. The pooled data showed that the neutralization of IL-17A significantly reduced the volume of large infarcts **(**Fig. 3A**)**. In contrast, an analysis of small infarcts showed no trend towards smaller infarct volumes in the anti-IL-17A group **(**Fig. 3B**)**.

One of the main effects of IL-17A in infections and sterile immune responses is to induce the expression of neutrophil attracting CXC-chemokines at the lesion site .^14^ The finding that we only detected a treatment effect of IL-17A neutralization in large infarcts with cortical involvement raised the question whether the IL-17A—neutrophil axis is predominantly activated in cortical areas.

**Figure 4:**
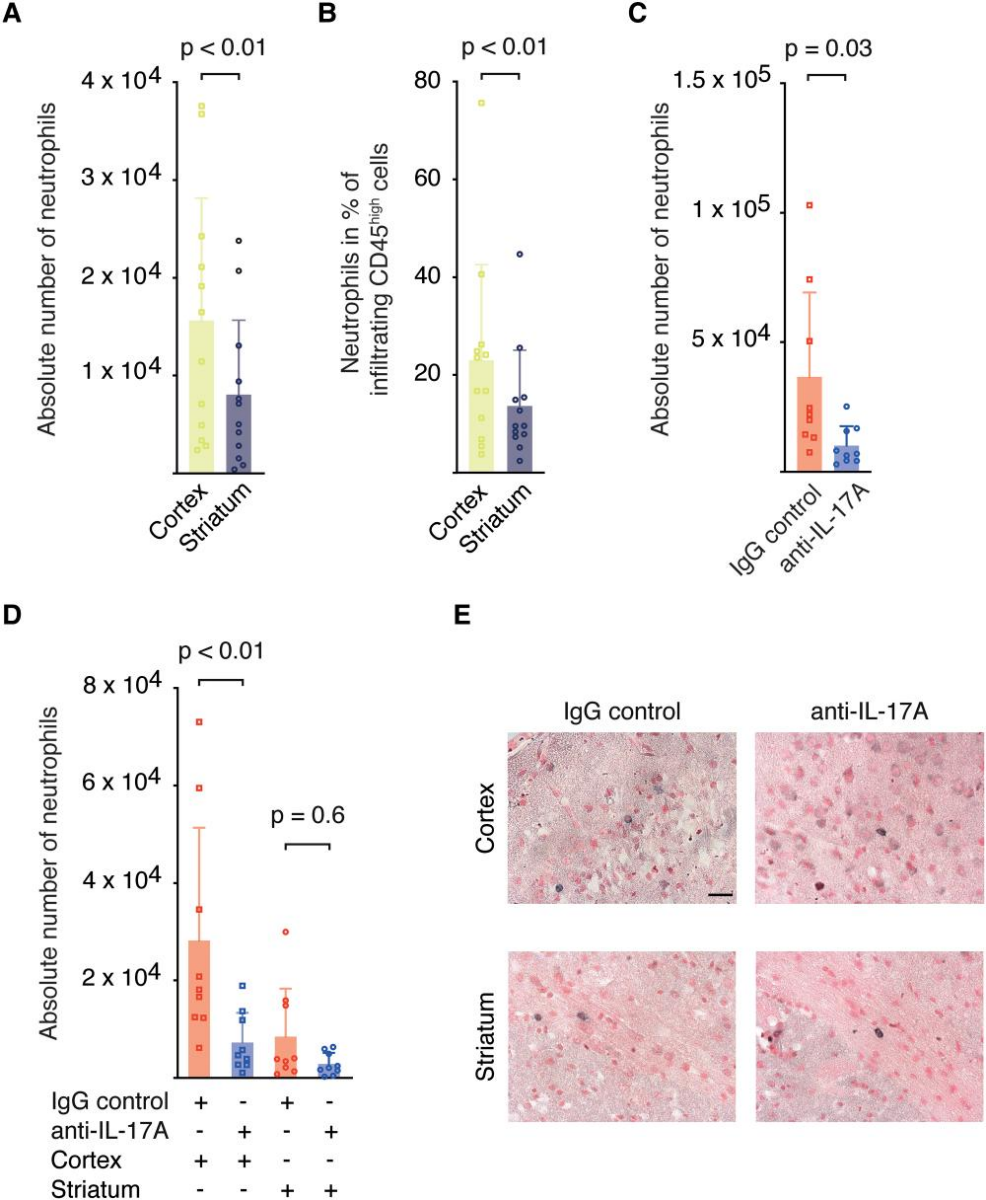
**IL-17A neutralization reduced neutrophil infiltration into the cortex.** (**A)** Absolute numbers of neutrophils and **(B)** percentages of neutrophils of all infiltrating CD45^high^ cells in cortical and striatal brain tissue three days following tMCAO of C57BL/6 mice. Graphs show mean ± SD. Statistical significance was assessed by Student’s *t*-test for dependent variables, **(A)***t*_(11)_ = 3.68, *P* < 0.01, *n* = 12; **(B)***t*_(11)_ = 3.8, *P* < 0.01, *n* = 12. Cell counts were determined by flow cytometry. **(C, D)** Ly6G^+^ cell counts per ischaemic hemisphere in the entire hemisphere **(C)** and divided into cortex and striatum **(D)** in representative anti-IL-17A (*n* = 9) and IgG control (*n* = 9) treated animals of our trial. Brains were processed for immunohistochemistry following MRI. Graphs show mean ± SD. Statistical analysis was performed with a two-factorial analysis of variances with repeated measures and Šidák’s multiple comparison test for pairwise comparisons (global: *F*(1,16) = 5.65, *P* = 0.03; cortex: *t*_(32)_ = 3.41, *P* < 0.01; striatum: *t*_(32)_ = 0.92, *P* = 0.6; *n* = 18). **(E)** Immunohistochemical staining of neutrophils (Ly6G^+^) in the cortex and striatum of mice from both treatment groups three days after tMCAO (scale bar 10 µm).

To answer this question, we separately analysed the neutrophil infiltration into the cortex and the striatum three days after tMCAO by flow cytometry in an independent cohort (Hamburg). The analysis revealed significantly higher absolute numbers of infiltrating neutrophils in cortical areas than in the striatum **(**Fig. 4A, Supplementary Fig. 6**)**, whereas absolute numbers of dendritic cells, macrophages, B cells, γδ T cells, Natural killer cells (NK cells), CD4^+^ and CD8^+^ T cells did not differ between cortex and striatum **(**Supplementary Fig. 6**)**. Consistent with the absolute cell counts, the proportion of neutrophils in the infiltrating CD45^high^ immune cells was significantly higher in cortical areas than in the striatum **(**Fig. 4B**)**, while the proportions of macrophages, CD4^+^ and CD8^+^ T cells were lower in cortical areas when compared with the striatum **(**Supplementary Figs 7 and 8**)**.

Next, we investigated the effect of IL-17A neutralization on neutrophil infiltration in brain tissue from a subset of mice from our multi-centre trial. Following MRI measurements, brains were sectioned, and absolute neutrophil numbers were quantified by immunohistochemical Ly6G staining. First, we observed that neutralization of IL-17A led to a significant reduction in the absolute number of neutrophils in the ischaemic hemisphere **(**Fig. 4C**)**. Second, in line with the flow cytometry data, we saw in the IgG control group that neutrophils predominantly migrated into cortical areas. When comparing the anti-IL-17A with the IgG control group, we found a highly significant inhibitory effect of anti-IL-17A treatment on neutrophil infiltration in the cortex but not the striatum **(**Fig. 4D and E**)**.

In summary, our data suggest, that the IL-17A—neutrophil axis is predominantly activated in large infarcts with involvement of cortical areas. A spatial activation of the IL-17A axis at the meningeal–cortical interface could also explain the treatment effect in large infarcts, whereas lesion size of small infarcts was not reduced by IL-17A neutralization.

### The gut microbiome differs between the four centres

**Figure 5:**
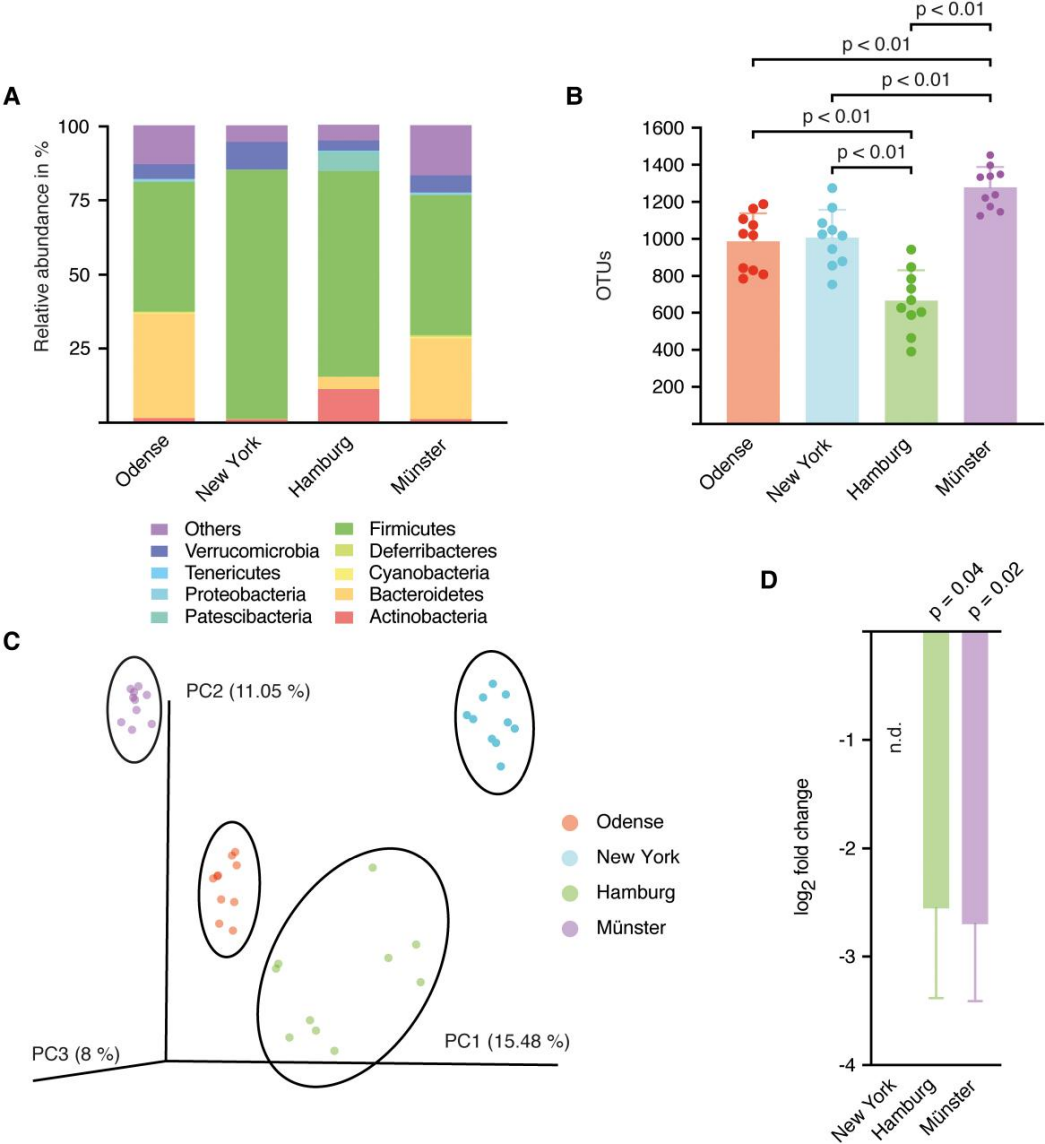
**The microbiome differed between the four centres.** (**A)** Taxonomic summary of OTUs on phylum level, *n* = 10 from each centre. **(B)** Total numbers of OTUs for each centre. Graphs show mean ± SD. Statistical significance was assessed by pairwise Student’s *t*-tests for independent samples (New York versus Odense: *t* = 0.29, *P* = 1; New York versus Hamburg, *t* = 4.76, *P* = 0.001; New York versus Münster: *t* = −4.55, *P* = 0.004; Hamburg versus Odense: *t* = −4.48, *P* = 0.002; Hamburg versus Münster: *t* = −9.65, *P* < 0.001; Münster versus Odense: *t* = −4.88, *P* = 0.001; *n* = 10 in each centre). **(C)** Principal coordinate analysis (PCoA) of the family composition. Clusters of each centre are highlighted with an ellipse. Statistical testing was performed with an analysis of similarities (*R* = 0.78, *P* < 0.001, *n* = 40). **(D)** qPCR analysis for SFB presence in mice from the four different centres. Expression levels were normalized to corresponding SFB levels in mice from Odense. Log_2_ fold changes are shown as mean ± SD. SFB were not detectable in the New York cohort. Statistical significance was assessed by Student’s *t*-tests for independent samples (Hamburg: *t*_(14)_ = 2.23, *P* = 0.04, *n* = 16; Münster: *t*_(16)_ = 2.58; *P* = 0.02, *n* = 18).

Interactions between the microbiota and the immune system affect the outcome following stroke.^26^ To evaluate any centre-specific differences in the composition of the microbiome with a possible influence on stroke outcome, we sequenced the variable regions v3 and v4 of the 16S rRNA of the gut bacteria. Specimens of 10 specific pathogen free mice of each centre were collected. The analysis of the OTUs revealed significant differences in the species diversity between most study centres **(**Fig. 5A and B**)**. Furthermore, the principal coordinate analysis (PCoA) showed distinct clusters, clearly distinguishing the four test centres **(**Fig. 5C**)**.

When analysing the effects of IL-17A neutralization in the individual centres, it became apparent that the anti-IL-17A treatment did not lead to a reduction in infarct size in one centre (Odense) **(**Supplementary Fig. 4A and B**).** In particular, the colonization with SFB can affect IL-17A levels in T cells. Therefore, we additionally quantified SFB in the faeces of animals from the different centres by RT-qPCR.^27^ Of note, animals from Odense had significantly higher amounts of SFB compared with Münster and Hamburg, while in New York no SFB were detectable **(**Fig. 5D**)**. Taken together, the microbiome and the SFB levels significantly differed between the four test centres, and colonization with SFB was associated with a reduced response to IL-17A neutralization.

## Discussion

Here, we report the results of an international multi-centre preclinical randomized and controlled trial on IL-17A neutralization in a murine ischaemia reperfusion stroke model. The objective of this pRCT was to evaluate short-term effects of IL-17A neutralization on infarct size three days after tMCAO. Using a precisely defined and uniform study protocol, this pRCT demonstrated that neutralization of IL-17A reduces infarct sizes and improves survival. These findings are confirmatory and verify results from previously published independent single-centre studies, which demonstrated the protective impact of IL-17A neutralization on stroke outcome.^13,14,19^ In the stroke field, our study is the third positive preclinical trial overall with an inflammatory target. Previously, treatment with anti-CD49d and IL-1 receptor antagonist showed beneficial effects in multi-centric preclinical studies.^10,11^

In response to the ‘replication crisis’, recent consensus statements emphasized the need for pRCTs to increase the translational impact of stroke research.^28^ Until today, most of the neuroprotective treatments which have shown to be effective in animal studies have failed in clinical trials.^3^ The design of preclinical studies is considered one of the main reasons for the failed translation. Experimental studies are often monocentric, whereas Phase III human trials are multi-centric.^29^ Common limitations of single-centre studies are inadequate statistical and operational study design and poor reporting.^30^ However, these limitations are not unique to neurovascular research.^3^ Other fields in biomedical science including neurodegenerative diseases,^31^ spinal cord injury^32^ and cancer^33,34^ are equally affected by the translational road block. The dilemma is illustrated by a recently published reproducibility project in the cancer field. By repeating 50 experiments from 23 papers, the authors showed that median effect sizes in the replication experiments were 85% smaller than in the original experiments.^34^

One measure to overcome limitations of preclinical *in vivo* studies is to conduct rigorously reported international, randomized and blinded preclinical multi-centre studies analogous to human clinical Phase III trials.^29^ Multi-centre trials with standardized protocols including an *a priori* power analysis are not only increasing external validity and inherent test reproducibility, they are also enhancing internal validity and transparency and facilitate enrolment.^35-38^ The design of our trial closely followed these proposed guidelines and therefore represents an important contribution to bridging the gap between preclinical experimental studies and human clinical trials in the stroke field.^9,37^

The overall design, implementation and enrolment of pRCTs is challenging. In order to conduct our study successfully at the four study centres, we made the following concessions in the study design: (i) we defined infarct size instead of neurological outcome as primary outcome parameter and (ii) we did not analyse long-term outcome comparable to the modified Ranking Scale at 90 days following stroke which is the common primary endpoint in human Phase III trials. To meet these limitations in future pRCTs, neurological outcome at clinically meaningful time points like 90 days should be analysed to further increase the translational potential. However, in our case the *a priori* sample size calculation was based on previously published monocentric treatment effects on infarct volume at Day 3 after tMCAO.^14^ Consequently, our pRCT was not powered to assess effects of an IL-17A neutralization on long-term neurological outcome as it is standard practice in human stroke studies.^39^ Another major limitation of our study is the exclusive analysis of young male mice but not female, aged or co-morbid mice. Especially age and gender should be considered in future pRCTs, since both variables shape IL-17A responses.^40,41^ To account for these variables of high translational relevance and to demonstrate the efficacy of an IL-17A neutralization even in a heterogeneous study cohort, the design of future pRCTs should include animals of different sex, comorbidities and age.

By individually analysing the effects of IL-17A neutralization in the single centres, we found significant effects in one centre only. The significant effect in our study cohort of 136 mice is thus influenced by the overall sample size. This finding is not unexpected. Similar to our study, human randomized, controlled, multi-centre trials analyse the treatment effect on the primary outcome parameter in the entire study cohort and not in sub-groups.^42^

Over the last decades, experimental research decoded the major contribution of highly conserved pro-inflammatory cytokines to neuronal injury in stroke pathophysiology (reviewed by Iadecola *et al*. ^43^). However, recent studies emphasized the need to consider that most of these cytokines, including IL-17A, IL-1 and tumor necrosis factor (TNF), have pleiotropic functions in the brain beyond inflammation (reviewed by Salvador *et al*.^44^). For example, IL-17A^+^ γδ T cells populate the meningeal space under homeostatic conditions as part of the meningeal immunity.^45^ Interestingly, these meningeal γδ T cells are not only producing IL-17A in response to pathogens but provide basal IL-17A levels even in the absence of inflammation^46^ with effects on homeostatic behaviour and memory.^45,47^ Therefore, future studies should additionally examine possible pleiotropic effects of anti-IL-17A treatment to comprehensively understand the consequences of therapeutic neutralization.

The pleiotropy of IL-17A effects in the brain is likely explained by the nature of IL-17 receptor signalling. The net effect of IL-17 receptor activation strongly depends on concomitant signals from other cytokines and other contextual variables.^48^ During inflammation, IL-17A synergizes with strong NF-κB activators, such as TNF, which is rapidly upregulated in microglia and macrophages after stroke.^49,50^ Thus, the pro-inflammatory effects of IL-17A in the ischaemic hemisphere depend on cooperative signalling with TNF.^14^ In line with the effects of IL-17A in acute immune defences, we demonstrated in our pRCT that the neutralization of IL-17A reduced migration of neutrophils into the ischaemic brain tissue. Interestingly, IL-17A neutralization only reduced neutrophil infiltration into the cortical tissue, whereas the recruitment into striatal areas was considerably low and not affected by anti-IL-17A treatment. The exclusive effect of IL-17A neutralization on cortical neutrophil infiltration might be, in part, explained by the localization of γδ T cells in the meningeal space, in direct proximity to the cortex, whereas γδ T cells are absent in sub-cortical areas and the striatum under homeostatic conditions.^19,45,47^ Another reason for the predominant accumulation of neutrophils in the ischaemic cortex may be the entry route of neutrophils via the leptomeninges. It was shown in the tMCAO model that neutrophils accumulate in the leptomeninges within the first 24 h after ischaemia.^51^ From the leptomeninges, neutrophils may be able to reach the ischaemic cortex via the subpial space and perivascular spaces of cortical vessels.^52,53^

Microbial cues have been previously reported to regulate the development and function of IL-17A^+^ T cell subsets in diverse tissues, including the gut and the meninges (reviewed by Papotto *et al*.^54^). Particularly, SFB shape the polarization of IL-17A^+^ T cells. Accordingly, Ivanov *et al*.^27^ observed a shift towards increased IL-17A levels in the gut of mice colonized by SFB. Of note, alterations of the gut microbiota also affect stroke outcome via IL-17A^+^ γδ T cells. In a murine stroke model, intestinal dysbiosis led to a shift in IL-17A levels of meningeal γδ T cells.^19^ Consistent with our data, the aforementioned study showed that a reduction in IL-17A^+^ γδ T cells had a protective effect on neurological outcome after experimental stroke. In the present pRCT, we observed substantial heterogeneity of the gut microbiome among the four participating centres, reflecting the heterogeneity in human cohorts.^55^ Thus, one can speculate that the heterogeneity of the microbiome in general and the frequency of SFB in particular contribute to the variance in stroke sizes and mortality between the four different centres in our pRCT. Since we did not determine IL-17A levels of γδ T cells in the individual centres within the framework of the pRCT, our data on the composition of the microbiome and the response to anti-IL-17A treatment are observational at this point. Therefore, further studies are needed to decipher the influence of the microbiome on the post-ischaemic inflammatory response and the response to IL-17A neutralization.

The last 20 years have seen two milestones in acute stroke treatment: intravenous (i.v.) thrombolysis^56^ and more recently endovascular thrombectomy.^57^ Although restoration of blood flow is crucial for the successful treatment of large vessel occlusions, many patients still present with clinically severe deficits even after successful recanalization. In this respect, it is necessary to develop new therapeutic targets that modulate the post-ischaemic tissue injury and complement recanalizing therapies. This pRCT offers promising evidence that IL-17A may be such an immunomodulatory therapeutic target.

In conclusion, in this randomized trial, anti-IL-17A treatment reduced infarct sizes and mortality in young male mice. In addition, we demonstrated the feasibility of preclinical randomized controlled stroke trials for novel therapeutic targets.

## Supplementary Material

fcad090_Supplementary_Data

## Abbreviations

ADC =: apparent diffusion coefficient
ARRIVE guidelines =: Animal Research: Reporting of In Vivo Experiments guidelines
DWI =: diffusion-weighted images
IgP =: immunoglobulin
IL-1 =: interleukin-1
IL-23 =: interleukin-23
IL-17A =: interleukin-17A
MCA =: middle cerebral artery
OTU =: operational taxonomic unit
PBS =: phosphate-buffered saline
PCoA =: principal coordinate analysis
PFA =: paraformaldehyde
pRCT =: preclinical randomised controlled trial
QIIME =: Quantitative Insights Into Microbial Ecology
RRID =: research resource identifiers
SFB =: segmented filamentous bacteria
tMCAO =: transient middle cerebral artery occlusion
TNF =: tumor necrosis factor
TTC =: 2,3,5-triphenyl-2-hydroxy-tetrazolium chloride

## References

[1] GBD 2016 Stroke Collaborators . Global, regional, and national burden of stroke, 1990-2016: A systematic analysis for the Global Burden of Disease Study 2016. Lancet Neurol. 2019;18(5):439–458. 30871944 10.1016/S1474-4422(19)30034-1 PMC6494974

[2] Schmidt-Pogoda A , Bonberg N, Koecke MHM, et al Why most acute stroke studies are positive in animals but not in patients: A systematic comparison of preclinical, early phase, and phase 3 clinical trials of neuroprotective agents. Ann Neurol. 2020;87(1):40–51. 31714631 10.1002/ana.25643

[3] O'Collins VE , Macleod MR, Donnan GA, Horky LL, van der Worp BH, Howells DW. 1,026 experimental treatments in acute stroke. Ann Neurol. 2006;59(3):467–477. 16453316 10.1002/ana.20741

[4] Baker M . 1,500 scientists lift the lid on reproducibility. Nature. 2016;533(7604):452–534. 27225100 10.1038/533452a

[5] Elkind MSV , Veltkamp R, Montaner J, et al Natalizumab in acute ischemic stroke (ACTION II): A randomized, placebo-controlled trial. Neurology. 2020;95(8):e1091–e1104. 32591475 10.1212/WNL.0000000000010038 PMC7668547

[6] Endres M , Moro MA, Nolte CH, Dames C, Buckwalter MS, Meisel A. Immune pathways in etiology, acute phase, and chronic sequelae of ischemic stroke. Circ Res. 2022;130(8):1167–1186. 35420915 10.1161/CIRCRESAHA.121.319994

[7] Percie du Sert N , Ahluwalia A, Alam S, et al Reporting animal research: Explanation and elaboration for the ARRIVE guidelines 2.0. PLoS Biol. 2020;18(7):e3000411. 32663221 10.1371/journal.pbio.3000411 PMC7360025

[8] Landis SC , Amara SG, Asadullah K, et al A call for transparent reporting to optimize the predictive value of preclinical research. Nature. 2012;490(7419):187–191. 23060188 10.1038/nature11556 PMC3511845

[9] Dirnagl U , Hakim A, Macleod M, et al A concerted appeal for international cooperation in preclinical stroke research. Stroke. 2013;44(6):1754–1760. 23598526 10.1161/STROKEAHA.113.000734 PMC3933930

[10] Llovera G , Hofmann K, Roth S, et al Results of a preclinical randomized controlled multicenter trial (pRCT): Anti-CD49d treatment for acute brain ischemia. Sci Transl Med. 2015;7(299):299ra121. 10.1126/scitranslmed.aaa9853 26246166

[11] Maysami S , Wong R, Pradillo JM, et al A cross-laboratory preclinical study on the effectiveness of interleukin-1 receptor antagonist in stroke. J Cereb Blood Flow Metab. 2016;36(3):596–605. 26661169 10.1177/0271678X15606714 PMC4776311

[12] Chamorro A , Dirnagl U, Urra X, Planas AM. Neuroprotection in acute stroke: Targeting excitotoxicity, oxidative and nitrosative stress, and inflammation. Lancet Neurol. 2016;15(8):869–881. 27180033 10.1016/S1474-4422(16)00114-9

[13] Shichita T , Sugiyama Y, Ooboshi H, et al Pivotal role of cerebral interleukin-17-producing gammadeltaT cells in the delayed phase of ischemic brain injury. Nat Med. 2009;15(8):946–950. 19648929 10.1038/nm.1999

[14] Gelderblom M , Weymar A, Bernreuther C, et al Neutralization of the IL-17 axis diminishes neutrophil invasion and protects from ischemic stroke. Blood. 2012;120(18):3793–3802. 22976954 10.1182/blood-2012-02-412726

[15] Gelderblom M , Gallizioli M, Ludewig P, et al IL-23 (interleukin-23)-producing conventional dendritic cells control the detrimental IL-17 (interleukin-17) response in stroke. Stroke. 2018;49(1):155–164. 29212740 10.1161/STROKEAHA.117.019101

[16] Schadlich IS , Vienhues JH, Jander A, et al Interleukin-1 mediates ischemic brain injury via induction of IL-17A in gammadelta T cells and CXCL1 in astrocytes. Neuromolecular Med. 2022;24:437–451. 35384588 10.1007/s12017-022-08709-y PMC9684245

[17] Veldhoen M . Interleukin 17 is a chief orchestrator of immunity. Nat Immunol. 2017;18(6):612–621. 28518156 10.1038/ni.3742

[18] Dai Q , Han S, Liu T, et al IL-17A neutralization improves the neurological outcome of mice with ischemic stroke and inhibits caspase-12-dependent apoptosis. Front Aging Neurosci. 2020;12:274. 33101005 10.3389/fnagi.2020.00274 PMC7500152

[19] Benakis C , Brea D, Caballero S, et al Commensal microbiota affects ischemic stroke outcome by regulating intestinal gammadelta T cells. Nat Med. 2016;22(5):516–523. 27019327 10.1038/nm.4068 PMC4860105

[20] Lee J , d'Aigle J, Atadja L, et al Gut Microbiota-derived short-chain fatty acids promote poststroke recovery in aged mice. Circ Res. 2020;127(4):453–465. 32354259 10.1161/CIRCRESAHA.119.316448 PMC7415518

[21] Gelderblom M , Leypoldt F, Steinbach K, et al Temporal and spatial dynamics of cerebral immune cell accumulation in stroke. Stroke. 2009;40(5):1849–1857. 19265055 10.1161/STROKEAHA.108.534503

[22] Schindelin J , Arganda-Carreras I, Frise E, et al Fiji: An open-source platform for biological-image analysis. Nat Methods. 2012;9(7):676–682. 22743772 10.1038/nmeth.2019 PMC3855844

[23] Gerriets T , Stolz E, Walberer M, et al Noninvasive quantification of brain edema and the space-occupying effect in rat stroke models using magnetic resonance imaging. Stroke. 2004;35(2):566–571. 14739415 10.1161/01.STR.0000113692.38574.57

[24] Lamprecht P , Fischer N, Huang J, et al Changes in the composition of the upper respiratory tract microbial community in granulomatosis with polyangiitis. J Autoimmun. 2019;97:29–39. 30420263 10.1016/j.jaut.2018.10.005

[25] Bolyen E , Rideout JR, Dillon MR, et al Author correction: Reproducible, interactive, scalable and extensible microbiome data science using QIIME 2. Nat Biotechnol. 2019;37(9):1091. 10.1038/s41587-019-0252-6 31399723

[26] Xia GH , You C, Gao XX, et al Stroke dysbiosis Index (SDI) in gut microbiome are associated with brain injury and prognosis of stroke. Front Neurol. 2019;10:397. 31068891 10.3389/fneur.2019.00397 PMC6491752

[27] Ivanov II , Atarashi K, Manel N, et al Induction of intestinal Th17 cells by segmented filamentous bacteria. Cell. 2009;139(3):485–498. 19836068 10.1016/j.cell.2009.09.033 PMC2796826

[28] Dirnagl U , Endres M. Found in translation: Preclinical stroke research predicts human pathophysiology, clinical phenotypes, and therapeutic outcomes. Stroke. 2014;45(5):1510–1518. 24652307 10.1161/STROKEAHA.113.004075

[29] Llovera G , Liesz A. The next step in translational research: Lessons learned from the first preclinical randomized controlled trial. J Neurochem. 2016;139(Suppl 2):271–279. 26968835 10.1111/jnc.13516

[30] Ioannidis JPA , Kim BYS, Trounson A. How to design preclinical studies in nanomedicine and cell therapy to maximize the prospects of clinical translation. Nat Biomed Eng. 2018;2(11):797–809. 30931172 10.1038/s41551-018-0314-y PMC6436641

[31] Perrin S . Preclinical research: Make mouse studies work. Nature. 2014;507(7493):423–425. 24678540 10.1038/507423a

[32] Steward O , Popovich PG, Dietrich WD, Kleitman N. Replication and reproducibility in spinal cord injury research. Exp Neurol. 2012;233(2):597–605. 22078756 10.1016/j.expneurol.2011.06.017

[33] Errington TM , Denis A, Perfito N, Iorns E, Nosek BA. Challenges for assessing replicability in preclinical cancer biology. Elife. 2021;10:e67995 34874008 10.7554/eLife.67995 PMC8651289

[34] Errington TM , Mathur M, Soderberg CK, et al Investigating the replicability of preclinical cancer biology. Elife. 2021:10:e71601. 34874005 10.7554/eLife.71601 PMC8651293

[35] Bath PMW , Macleod MR, Green AR. Emulating multicentre clinical stroke trials: A new paradigm for studying novel interventions in experimental models of stroke. Int J Stroke. 2009;4(6):471–479. 19930059 10.1111/j.1747-4949.2009.00386.x

[36] Maertens O , McCurrach ME, Braun BS, et al A collaborative model for accelerating the discovery and translation of cancer therapies. Cancer Res. 2017;77(21):5706–5711. 28993414 10.1158/0008-5472.CAN-17-1789 PMC5668167

[37] Dirnagl U , Fisher M. International, multicenter randomized preclinical trials in translational stroke research: It's time to act. J Cereb Blood Flow Metab. 2012;32(6):933–935. 22510602 10.1038/jcbfm.2012.51 PMC3367233

[38] Drude NI , Martinez Gamboa L, Danziger M, Dirnagl U, Toelch U. Improving preclinical studies through replications. Elife. 2021;10:e62101. 33432925 10.7554/eLife.62101 PMC7817176

[39] Thomalla G , Simonsen CZ, Boutitie F, et al MRI-guided thrombolysis for stroke with unknown time of onset. N Engl J Med. 2018;379(7):611–622. 29766770 10.1056/NEJMoa1804355

[40] Chen HC , Eling N, Martinez-Jimenez CP, et al IL-7-dependent compositional changes within the gammadelta T cell pool in lymph nodes during ageing lead to an unbalanced anti-tumour response. EMBO Rep. 2019;20(8):e47379. 31283095 10.15252/embr.201847379 PMC6680116

[41] Luo X , Chen O, Wang Z, et al IL-23/IL-17A/TRPV1 axis produces mechanical pain via macrophage-sensory neuron crosstalk in female mice. Neuron. 2021;109(17):2691–2706 e5. 34473953 10.1016/j.neuron.2021.06.015 PMC8425601

[42] Moher D , Hopewell S, Schulz KF, et al CONSORT 2010 explanation and elaboration: Updated guidelines for reporting parallel group randomised trials. BMJ. 2010;340:c869. 20332511 10.1136/bmj.c869 PMC2844943

[43] Iadecola C , Buckwalter MS, Anrather J. Immune responses to stroke: Mechanisms, modulation, and therapeutic potential. J Clin Invest. 2020;130(6):2777–2788. 32391806 10.1172/JCI135530 PMC7260029

[44] Salvador AF , de Lima KA, Kipnis J. Neuromodulation by the immune system: A focus on cytokines. Nat Rev Immunol. 2021;21(8):526–541. 33649606 10.1038/s41577-021-00508-z

[45] Ribeiro M , Brigas HC, Temido-Ferreira M, et al Meningeal gammadelta T cell-derived IL-17 controls synaptic plasticity and short-term memory. Sci Immunol. 2019;4:40. 10.1126/sciimmunol.aay5199 PMC6894940 31604844

[46] Rustenhoven J , Drieu A, Mamuladze T, et al Functional characterization of the dural sinuses as a neuroimmune interface. Cell. 2021;184(4):1000–1016 e27. 33508229 10.1016/j.cell.2020.12.040 PMC8487654

[47] Alves de Lima K , Rustenhoven J, Da Mesquita S, et al Meningeal gammadelta T cells regulate anxiety-like behavior via IL-17a signaling in neurons. Nat Immunol. 2020;21:1421–1429. 32929273 10.1038/s41590-020-0776-4 PMC8496952

[48] Li X , Bechara R, Zhao J, McGeachy MJ, Gaffen SL. IL-17 receptor-based signaling and implications for disease. Nat Immunol. 2019;20(12):1594–1602. 31745337 10.1038/s41590-019-0514-y PMC6943935

[49] Clausen BH , Lambertsen KL, Dagnaes-Hansen F, et al Cell therapy centered on IL-1Ra is neuroprotective in experimental stroke. Acta Neuropathol. 2016;131(5):775–791. 26860727 10.1007/s00401-016-1541-5 PMC4835531

[50] Yli-Karjanmaa M , Clausen BH, Degn M, et al Topical administration of a soluble TNF inhibitor reduces infarct volume after focal cerebral ischemia in mice. Front Neurosci. 2019;13:781. 31440125 10.3389/fnins.2019.00781 PMC6692878

[51] Otxoa-de-Amezaga A , Gallizioli M, Pedragosa J, et al Location of neutrophils in different compartments of the damaged mouse brain after severe ischemia/reperfusion. Stroke. 2019;50(6):1548–1557. 31084324 10.1161/STROKEAHA.118.023837

[52] Zhang ET , Inman CB, Weller RO. Interrelationships of the pia mater and the perivascular (Virchow-Robin) spaces in the human cerebrum. J Anat. 1990;170:111–123. 2254158 PMC1257067

[53] Perez-de-Puig I , Miro-Mur F, Ferrer-Ferrer M, et al Neutrophil recruitment to the brain in mouse and human ischemic stroke. Acta Neuropathol. 2015;129(2):239–257. 25548073 10.1007/s00401-014-1381-0

[54] Papotto PH , Yilmaz B, Silva-Santos B. Crosstalk between gammadelta T cells and the microbiota. Nat Microbiol. 2021;6(9):1110–1117. 34341528 10.1038/s41564-021-00948-2

[55] Hall AB , Tolonen AC, Xavier RJ. Human genetic variation and the gut microbiome in disease. Nat Rev Genet. 2017;18(11):690–699. 28824167 10.1038/nrg.2017.63

[56] National Institute of Neurological Disorders and Stroke rt-PA Stroke Study Group . Tissue plasminogen activator for acute ischemic stroke. N Engl J Med. 1995;333(24):1581–1587. 7477192 10.1056/NEJM199512143332401

[57] Berkhemer OA , Fransen PSS, Beumer D, et al A randomized trial of intraarterial treatment for acute ischemic stroke. N Engl J Med. 2015;372(1):11–20. 25517348 10.1056/NEJMoa1411587

