# Supplementary material for: A preclinical randomized controlled multi-centre trial of anti-interleukin-17A treatment for acute ischaemic stroke
Source: Brain Commun. 2023 Mar 23;5(2):fcad090. doi: 10.1093/braincomms/fcad090 (PMC10088471; doi:10.1093/braincomms/fcad090)
Supplement: fcad090_Supplementary_Data [file fcad090_supplementary_data.pdf]

Supplemental Material (online data)

Study protocol distributed to the centers:

Preclinical randomized controlled multicenter trial to assess the efficiency of an anti-IL-17A antibody treatment in tMCAO

1. Background

Several studies in experimental stroke have shown that the disruption of the IL-17A axis is protective in stroke[1-3]. In murine models of stroke *Il17a*$^{-/-}$ and *Il17ra*$^{-/-}$ mice were protected from ischemic tissue damage and anti-IL-17A antibody treatments improved neurological outcome. Currently available studies were performed in single centers.

To overcome the low reproducibility and translation of preclinical single center studies preclinical randomized controlled multicenter trials (pRCT) were proposed. The aim of the current study is to test the efficiency of an anti-IL-17A antibody treatment in a preclinical randomized controlled multicenter stroke trial.

Our protocol fulfills fundamental requirements of pRCTs:

- harmonization of experimental protocols among study centers
- a priori sample size calculation
- randomization of the treatment groups
- blinding of all investigators with respect to treatment allocation
- centralized study organization
- analysis by an independent research center

2. Material provided by Hamburg

- anti-IL-17A antibody (aliquots à 500 µg (3.33 µg/µl), clone MM17F3, 16.6 mg/kg bodyweight)

- isotype-control antibody (IgG1) (aliquots à 500 µg (3.33 µg/µl), 16.6 mg/kg bodyweight)

- filament (see below)

3. Standardized tMCAO procedure

see: study design in Supplemental Figure 2

3.1. Randomization

Anti-IL-17A antibody and IgG control will be blinded and coded in Hamburg and distributed to each study center.

Antibody treatment, tMCAO, analysis of infarct volumes and secondary outcome parameters will be performed by researchers who are blinded with respect to the treatment groups. Unblinding will be performed after completion of the statistical analyses.

3.2. Power analysis

Power analysis on the basis of previously published effects of an anti-IL-17A treatment in comparison to an isotype antibody treatment results in:

t tests - Means: Wilcoxon-Mann-Whitney test (two groups)

Analysis: A priori: Compute required sample size

Input:	Tail(s)	= One
	Parent distribution	= Normal
	Effect size d	= 1,2
	α err prob	= 0,05
	Power (1-β err prob)	= 0,95
	Allocation ratio N2/N1	= 1
Output:	Noncentrality parameter δ	= 3,42
	Critical t	= 1,69
	Df	= 30,48
	**Sample size group 1**	**= 17**
	**Sample size group 2**	**= 17**
	Total sample size	= 34
	Actual power	= 0,9551119

3.3. Animals

- C57BL/6 mice
- males
- age 11-13 weeks
- weight 24-28 g
- strokes should be performed between 8.00 a.m. and 4 p.m.

3.4. anti-IL-17A or IgG control administration

- 500 µg / animal
- concentration of 500 µg in 150 µl
- 1h after reperfusion
- intravenous administration: retro-orbital or tail vein
- **group size:** **- anti-IL-17A 17 animals**
  - IgG control 17 animals

3.5. MCAO procedure

3.5.1 Filament:

- Silicon rubber-coated monofilament for tMCAO model

- Filament size 6-0, diameter 0.09-0.11 mm, length 20 mm, diameter with coating 0.21 ± 0.02 mm, and coating length 1.5-2 mm. Pack of 10 filaments. Doccol filaments: 6-0 fine MCAO suture L12PK10 (# 602112PK10)

- Filament will be provided by Hamburg

- Filaments can be re-used if not damaged

3.5.2. Occlusion of the left MCA

3.5.3. Occlusion time:

- 45 min

3.5.4. Temperature control of mice during surgery:

- 35.5-36.5 °C

3.5.5. Daily measurement of bodyweight

3.6. Monitoring during tMCAO (before removal of the filament)

3.6.1. If possible - physiological parameters:

- peripheral oxygen saturation
- heart rate
- breathing frequency

3.6.2. In every mouse laser doppler:

- blood flow contralateral / ipsilateral (before removal of the filament)
- only mice with a reduction of blood flow on the ipsilateral side ≥80% in comparison to the contralateral side are included

4. Neurological Scoring

4.1. Bederson Score

- 3h post MCAO
- 1d post MCAO
- 3d post MCAO

5. Collection of brains on day 3 post tMCAO

Mice will be scarified on day 3.

5.1. Mice will be perfused with:

- 25 ml PBS, 10 ml/min, 4°C
- 25 ml PFA, 10 ml/min, 4°C
- 24 h post fix in 4 % PFA in PBS, 4°C
- transfer of brains into PBS, 4°C

5.2. Cooled brains will be sent to HH.

6. Read out parameters

6.1. Primary read out parameter

Infarct volumes on day 3 post tMCAO will measured by diffusion weighted MRI (apparent diffusion coefficient (ADC)) in Hamburg (Supplemental Figure 1).

6.2. Secondary outcome parameters

6.2.1. Mortality

6.2.2. Neurological score

6.2.3. Histological analysis of the neutrophil infiltration in ipsilesional hemispheres

7. Exclusion criteria for analysis of the primary read out parameter

7.1 Preanalytical exclusion criteria

7.1.1. Death before reaching the primary endpoint

7.1.2. Body temperature outside the accepted range

7.1.3. Reduction of the laser doppler flow on the ipsilateral side <80% in comparison to the contralateral side

7.2 Analytical exclusion criteria

7.2.1 Significant destruction of the brain after removal

7.2.2 Visible parenchymal or subarachnoid hemorrhage

7.2.4. No visible infarct on the MRI

7.2.4 Isolated infarct of the brainstem

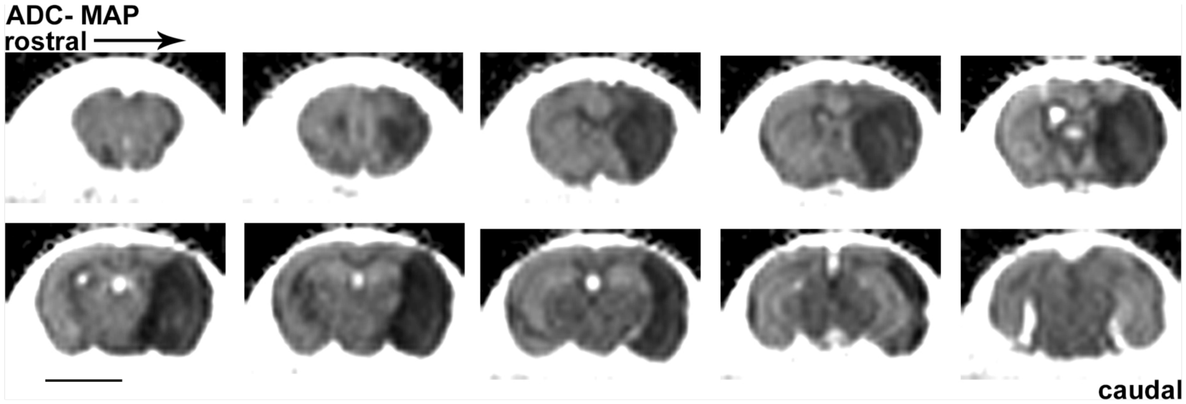

Supplemental Figure 1: Example of infarct size analysis by MRI (ADC) of brains fixed with 4% PFA d3 following tMCAO (scale bar 5 mm).

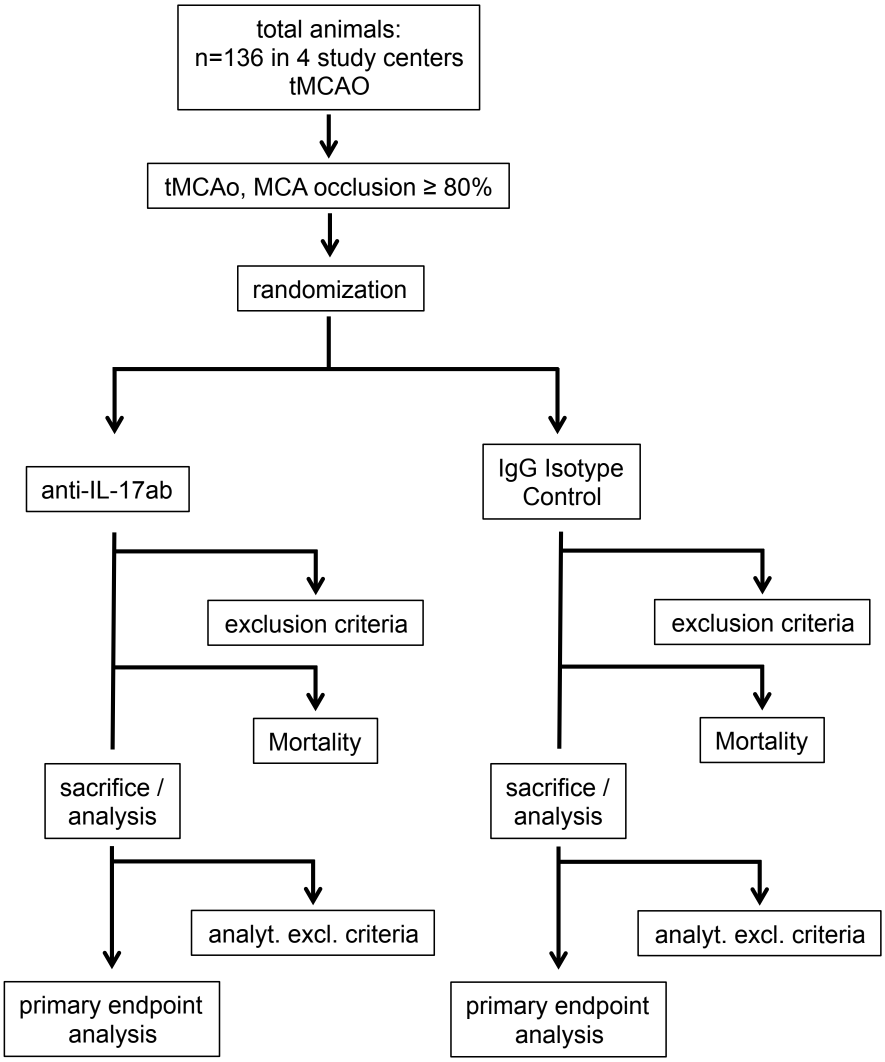

Supplemental Figure 2: Design of the preclinical randomized controlled multicenter trial.

Literature:

1. Shichita T, Sugiyama Y, Ooboshi H, Sugimori H, Nakagawa R, Takada I, Iwaki T, Okada Y, Iida M, Cua DJ, Iwakura Y, Yoshimura A. Pivotal role of cerebral interleukin-17-producing gammadeltaT cells in the delayed phase of ischemic brain injury. Nat Med. 2009; 15(8): 946-50.
2. Gelderblom M, Weymar A, Bernreuther C, Velden J, Arunachalam P, Steinbach K, Orthey E, Arumugam TV, Leypoldt F, Simova O, Thom V, Friese MA, Prinz I, Holscher C, Glatzel M, Korn T, Gerloff C, Tolosa E, Magnus T. Neutralization of the IL-17 axis diminishes neutrophil invasion and protects from ischemic stroke. Blood. 2012; 120(18): 3793-802.
3. Benakis C, Brea D, Caballero S, Faraco G, Moore J, Murphy M, Sita G, Racchumi G, Ling L, Pamer EG, Iadecola C, Anrather J. Commensal microbiota affects ischemic stroke outcome by regulating intestinal gammadelta T cells. Nat Med. 2016; 22(5): 516-23.

Supplemental Figure 3

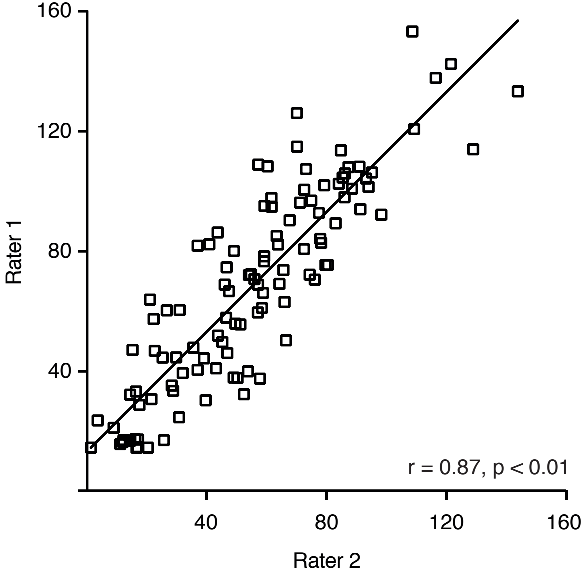

Supplemental Figure 3. Comparison of the volumetry of the raters.

Correlation of MRI based ratings of infarct volumes of rater one and two (Pearson r=0.87, CI=0.82 – 0.91, p<0.001, n=109 pairs).

Supplemental Figure 4

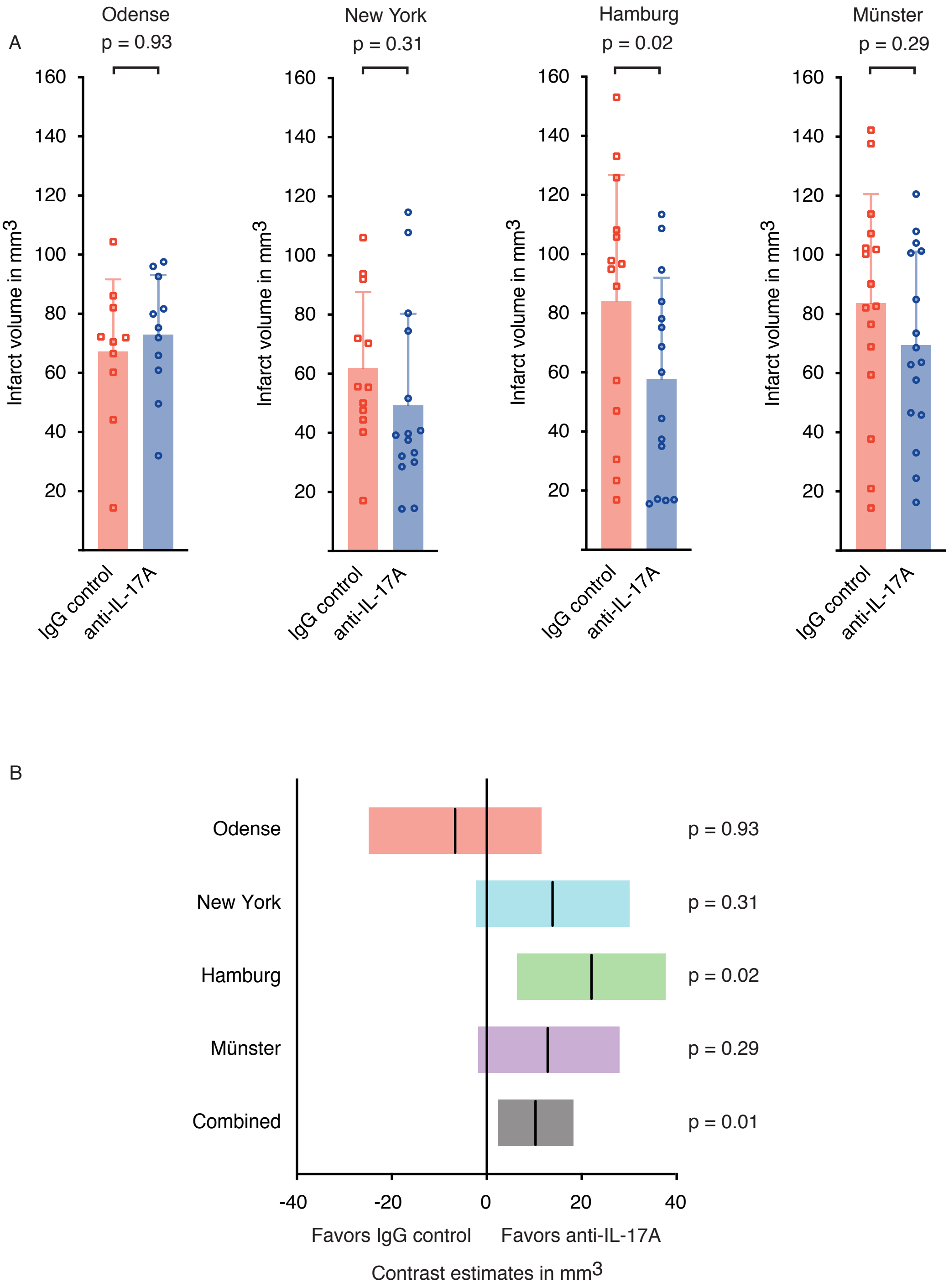

Supplemental Figure 4. Infarct sizes of individual centers.
(A) The infarct sizes of rater one in mm^3 for each individual center. Infarct volumes were analyzed three days following tMCAO by MRI. Graphs show mean±SDs. Statistical significance was assessed by linear-mixed model analysis with pairwise comparisons. **(B)** Pairwise comparisons of infarct volumes for single centers. Post-hoc Šidák adjustments were made for the alpha level. The estimated difference is shown in mm^3±95% CI (Odense: $F_{(1,209)}$=0.51, p=0.93, n=21; New York: $F_{(1,209)}$=3.04, p=0.31, n=27; Hamburg: $F_{(1,209)}$=7.76, p=0.02, n=29; Münster: $F_{(1,209)}$=2.88, p=0.29, n=32).

Supplemental Figure 5

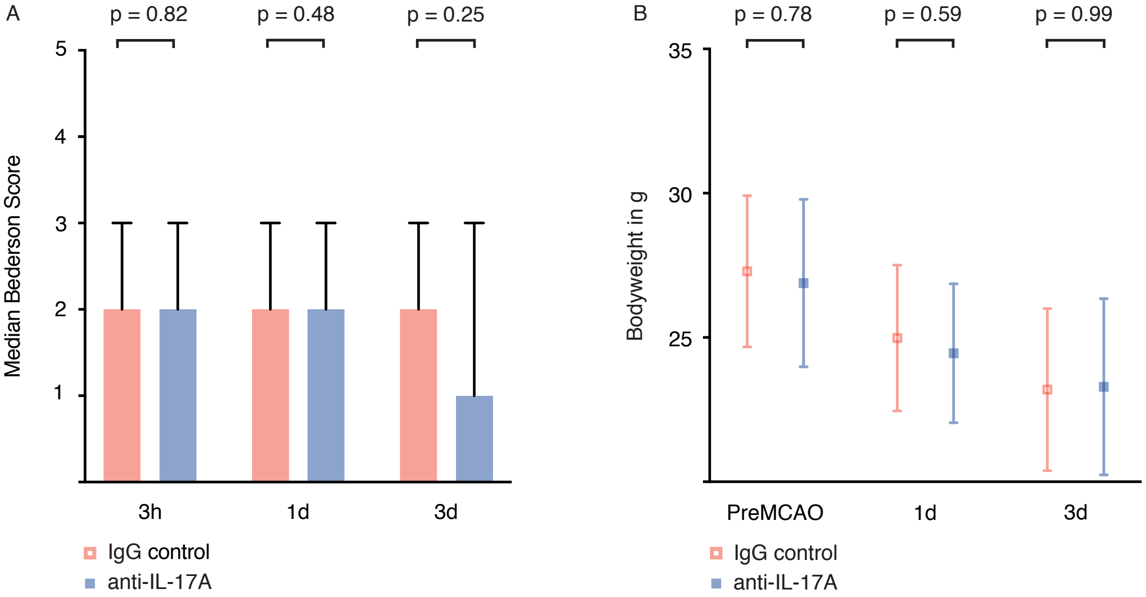

Supplemental Figure 5. Bederson neuroscore and bodyweight as secondary outcome parameters.
(A) Neurological scores at 3 h and at days 1 and 3 of animals that were treated with 500 *μ*g isotype control or 500 *μ*g of anti-IL-17A. Shown is the median with range. Significance was assessed by Mann-Whitney-U test (3 hours: U=1878, p=0.82, n=62 in both groups; day 1: U=1417, p=0.48, n= 59 in the anti-IL-17A group and n=52 in the IgG control group; day 3: U=1341, p=0.25, n=60 in the anti-IL-17A group and n=51 in the IgG control group). **(B)** Bodyweight in g of mice of both treatment groups. Statistical analysis was performed with a linear mixed model analysis and Šidák's multiple comparison tests for pairwise comparisons (preMCAO: $t_{(125.4)}$=0.84, p=0.78, n=128; day 1: $t_{(109.4)}$=1.14, p=0.59, n=114; day 3: $t_{(106.3)}$=0.17, p=0.99, n=109).

Supplemental Figure 6

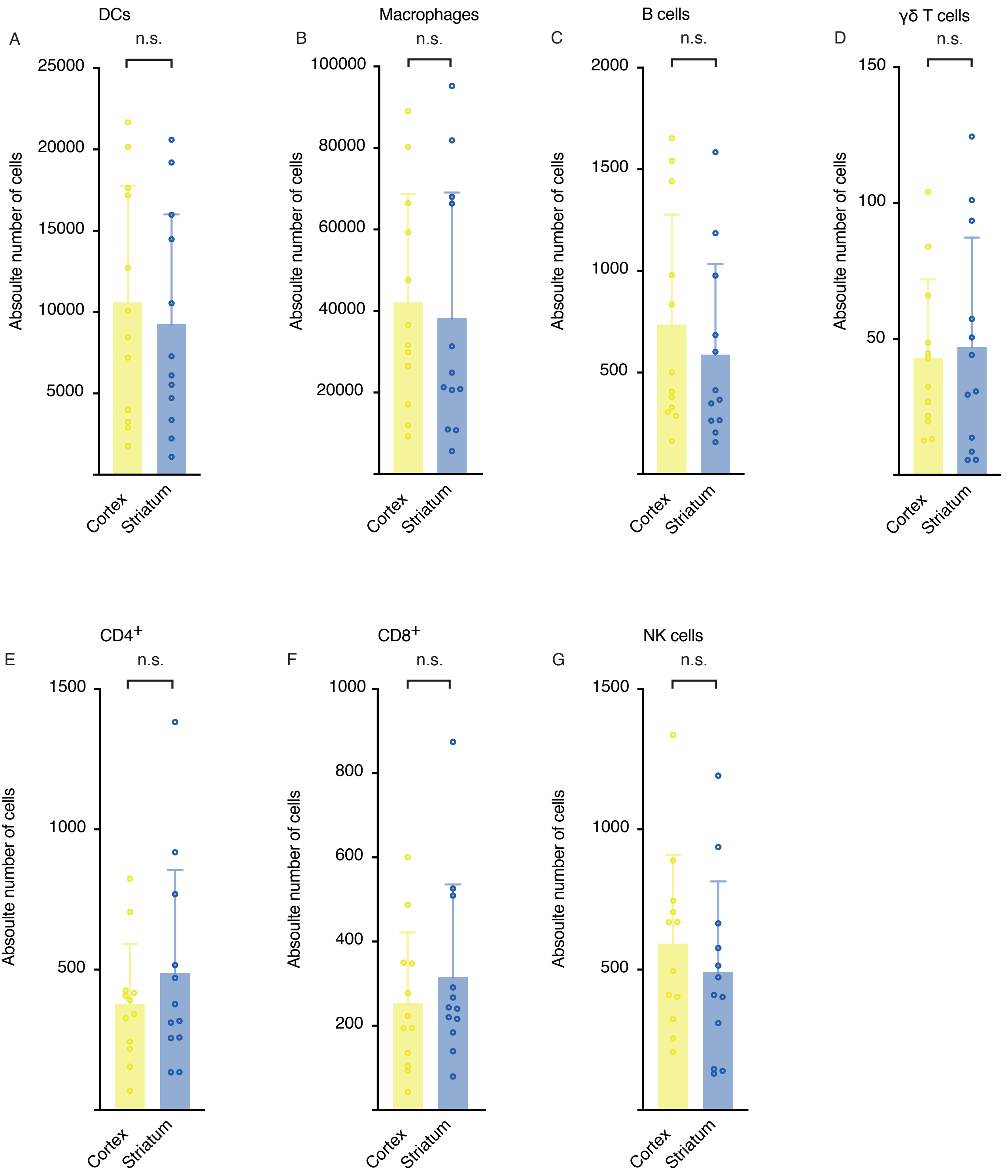

Supplemental Figure 6. Absolute numbers of brain-infiltrating immune cell subpopulations.
(A-G) Absolute numbers of brain-infiltrating immune cell subpopulations in cortical and striatal brain tissue per ischemic hemisphere three days following tMCAO. Graphs show mean ± SD. Statistical significance was assessed by Student's t-test for dependent variables. Cell counts were determined by flow cytometry. **(A)** $t_{(11)}$=0.81, p=0.43, n=12; **(B)** $t_{(11)}$=0.78, p=0.45, n=12; **(C)** $t_{(11)}$=1.77, p=0.10, n=12; **(D)** $t_{(11)}$=0.41, p=0.69, n=12; **(E)** $t_{(11)}$=1.47, p=0.17, n=12; **(F)** $t_{(11)}$=1.23, p=0.25, n=12; **(G)** $t_{(11)}$=1.64, p=0.13, n=12.

Supplemental Figure 7

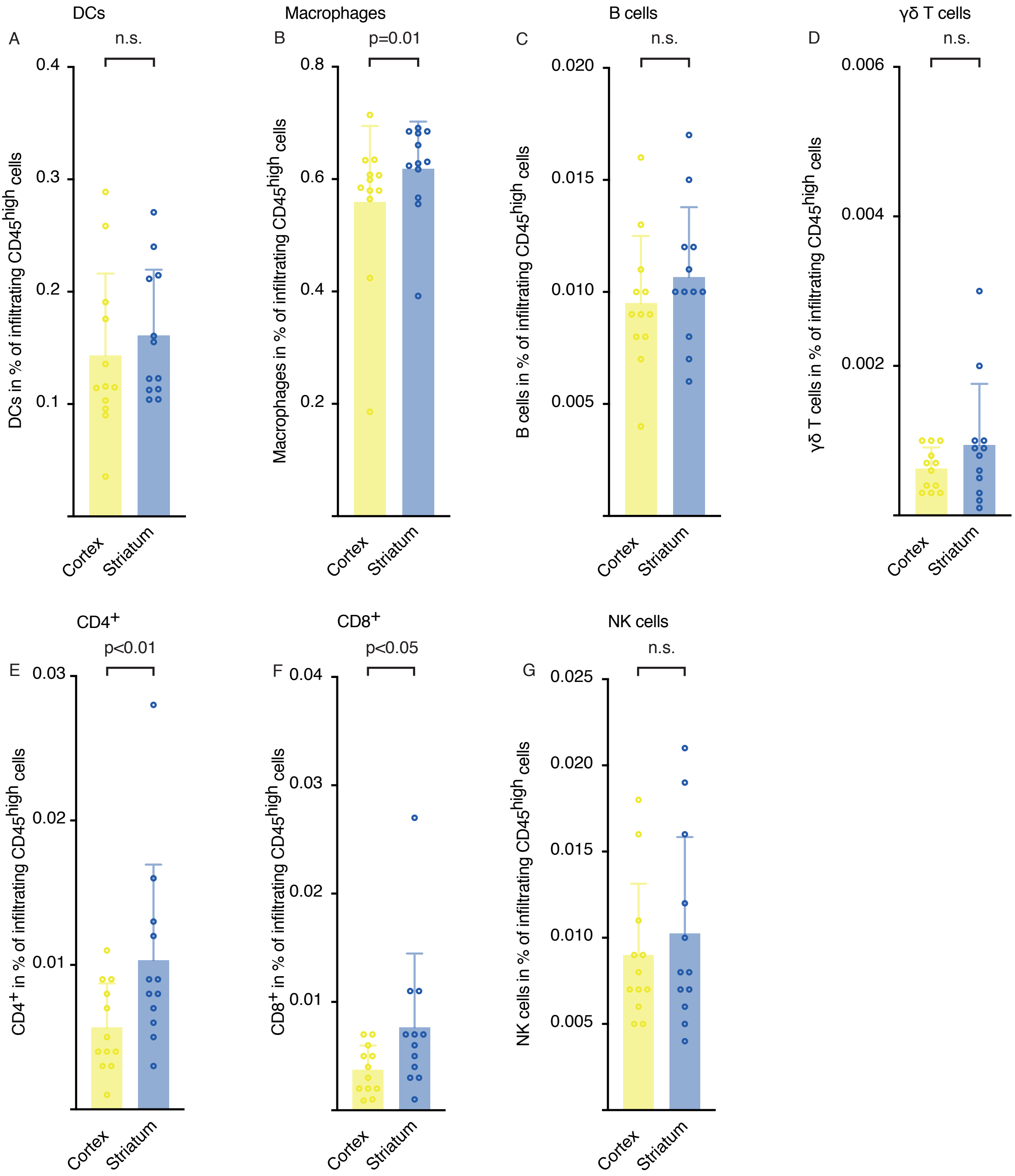

Supplemental Figure 7. Relative numbers of brain-infiltrating immune cell subpopulations.
(A-G) Relative numbers of brain-infiltrating immune cell subpopulations in cortical and striatal brain tissue per ischemic hemisphere three days following tMCAO. Graphs show mean ± SD. Statistical significance was assessed by Student's t-test for dependent variables. Cell counts were determined by flow cytometry. **(A)** $t_{(11)}$=2.01, p=0.07, n=12; **(B)** $t_{(11)}$=2.30, p=0.01, n=12; **(C)** $t_{(11)}$=1.20, p=0.26, n=12; **(D)** $t_{(11)}$=1.52, p=0.16, n=12; **(E)** $t_{(11)}$=3.71, p<0.01, n=12; **(F)** $t_{(11)}$=2.38, p=0.03, n=12; **(G)** $t_{(11)}$=1.39, p=0.19, n=12.

Supplemental Figure 8

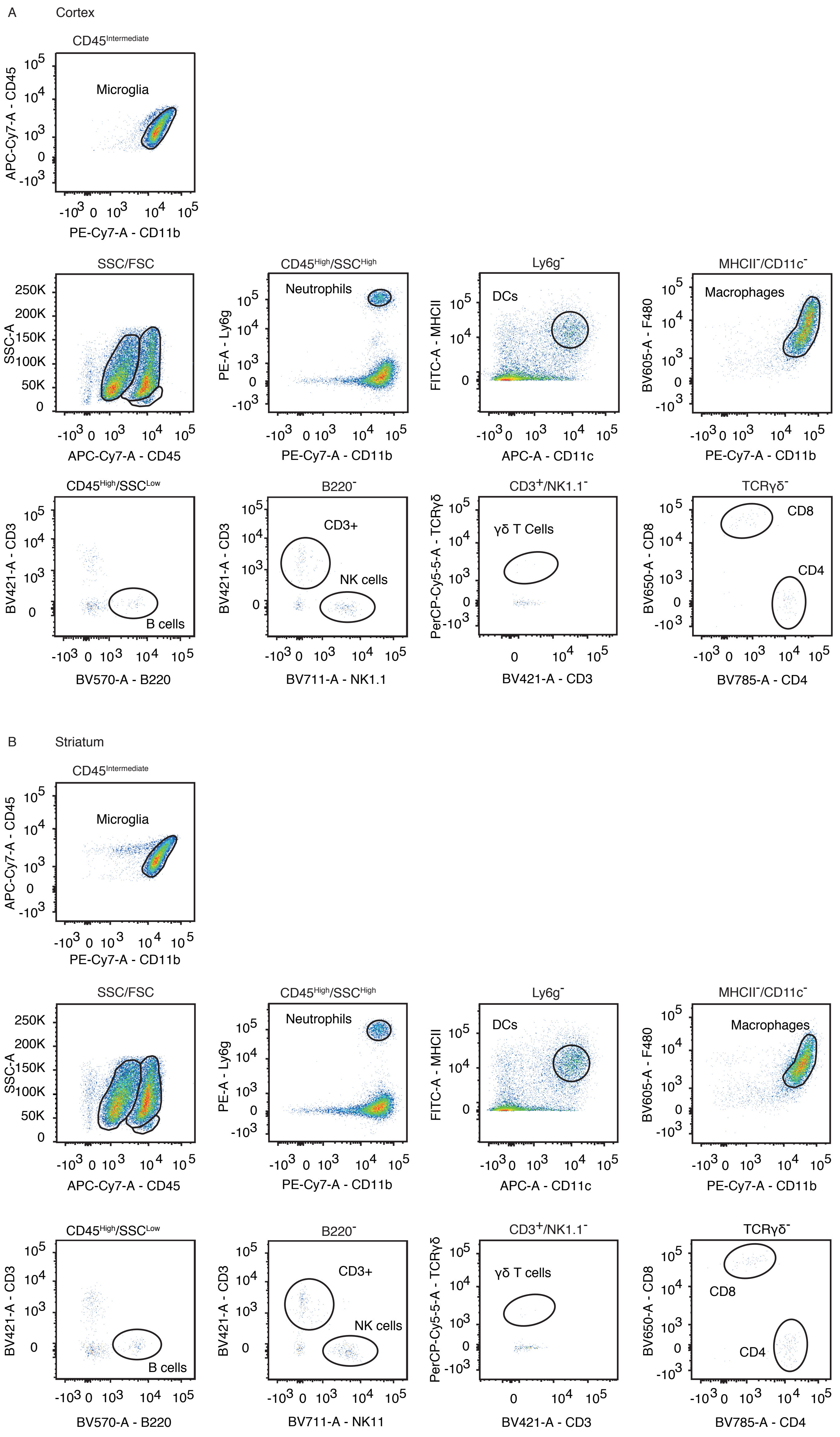

Supplemental Figure 8. Representative gating strategy for brain-infiltrating immune cell subpopulations. Flow cytometry of brain-infiltrating immune cell subpopulations from cortical **(A)** and striatal **(B)** brain tissue. Gating strategy to identify microglia, neutrophils, dendritic cells, macrophages, B cells, NK cells, γδ T cells, CD4 and CD8 T cells after staining for CD45, CD11b, Ly6g, CD11c, MHCII, F4/80, B220, CD3, NK1.1, TCRγδ, CD4 and CD8. FSC indicates forward scatter, SSC indicates side scatter.
